# Systematic analysis of ZDHHC9 as a potential prognostic and immunotherapy biomarker in breast cancer

**DOI:** 10.3389/fimmu.2025.1609621

**Published:** 2025-07-16

**Authors:** Desheng Zhou, Zhenpeng Wu, Yachao Cui, Doudou Wang, Hejun Song, Jun Chen, Hong Lin

**Affiliations:** ^1^ The Sixth School of Clinical Medicine, The Affiliated Qingyuan Hospital (Qingyuan People’s Hospital), Guangzhou Medical University, Qingyuan, China; ^2^ Guangzhou Institute of Cancer Research, The Affiliated Cancer Hospital, Guangzhou Medical University, Guangzhou, China; ^3^ The First Affiliated Hospital of Guangzhou Medical University, Guangzhou Institute of Respiratory Health, National Clinical Research Center for Respiratory Disease, State Key Laboratory of Respiratory Disease, Guangzhou, China; ^4^ Guangzhou National Laboratory, Guangzhou, China; ^5^ Shaanxi Energy Institute, Shaanxi, China; ^6^ Thyroid and Breast Surgery Department, Shenzhen Hospital, Southern Medicaid University, Shenzhen, China

**Keywords:** breast cancer, immunotherapy, prognosis, tumor microenvironment, Zdhhc9

## Abstract

**Background:**

Breast cancer (BC) represents a highly heterogeneous malignancy and continues to be a leading source of death among women worldwide. Enhancing diagnostic and therapeutic approaches necessitates a thorough grasp of the underlying molecular pathways and the identification of dependable biomarkers. Although palmitoyl transferases, particularly ZDHHC9, have been associated with the progression of various cancers, their specific role in BC remains incompletely understood.

**Methods:**

In this investigation, TCGA and GTEx databases were utilized to analyze the expression patterns of ZDHHC9 and to evaluate its prognostic significance. Moreover, the regulatory pathways involving ZDHHC9 were explored via co-expression analysis and differential gene enrichment studies. Insights into ZDHHC9 expression across different cell types and its potential oncogenic pathways were derived from scRNA sequencing analysis. Additionally, immunophenoscore (IPS), EaSIeR and immunotherapy cohorts were utilized to predict immunotherapy responses. The biological significance of ZDHHC9 was verified through *in vitro* and *in vivo* experiments.

**Results:**

Our findings revealed that ZDHHC9 is markedly overexpressed in BC, with elevated levels of ZDHHC9 being correlated with poor survival outcomes, suggesting its role as an independent risk factor in BC. Furthermore, high ZDHHC9 expression was found to be associated with multiple immune cell types within BC. Notably, patients exhibiting lower ZDHHC9 expression demonstrated a higher likelihood of benefitting from immunotherapy. Lastly, the vivo and vitro experiments consistently demonstrated that suppression of ZDHHC9 expression could reduce BC cell proliferation.

**Conclusions:**

This study highlights ZDHHC9 as a potential prognostic marker, a regulator of tumor immunity, and a biomarker of therapeutic response in BC, offering a promising avenue for improving BC diagnosis and treatment.

## 1 Introduction

As of 2022, breast cancer (BC) has been responsible for approximately 2.3 million new cases, establishing it among the most prevalent cancers globally, second only to lung cancer, representing about 11.6% of all cancer diagnoses (1). Among women, it remains the most frequently identified malignancy and remains the principal factor in cancer-related mortality, with a death rate of 15.4% (1). Although significant advancements have been achieved in surgical, chemotherapeutic, radiotherapeutic, endocrine, and targeted therapies, the mortality rate associated with advanced-stage BC remains alarmingly high (2). This persistent challenge is primarily attributed to the various regulatory molecular mechanisms of the occurrence and development of BC, which greatly affect the prognosis and treatment options of patients (3, 4). Consequently, further investigation into additional molecular mechanisms implicated in BC regulation is imperative for discovering novel diagnostic markers and treatment interventions.

S-palmitoylation, a reversible post-translational lipid modification, is widely observed in human cells, where it modulates protein localization, stability, and function (5). Palmitoyl transferases, classified under the zinc finger Asp-His-His-Cys-type (ZDHHC) family, encompass 23 distinct genes in mammals (ZDHHC1–ZDHHC24, with the exception of ZDHHC10) (6). Evidence indicates that these enzymes are involved in the regulation of tumor progression and the tumor immune microenvironment. It is reported that ZDHHC3 is markedly overexpressed in both malignant and metastatic BCs, and its knockdown in MDA-MB-231 cells triggers oxidative stress, senescence, and heightened recruitment of antitumor macrophages and natural killer cells (7). Additionally, ZDHHC3 ensures the palmitoylation of the cysteine residue on ITGβ4, thereby enhancing BC cell invasion (8). ZDHHC5, by regulating the palmitoylation of Flotillin2, contributes to the oncogenic activity of lipid rafts (9). ZDHHC9 facilitates the palmitoylation of PD-L1, allowing its association with PD-1 located on T cells to transmit immunosuppressive signals (10). However, the specific associations of the 23 identified palmitoyl transferases with BC, along with their potential to predict prognosis or function as therapeutic targets, remain to be fully elucidated. Consequently, determining the ZDHHC enzymes most relevant to BC treatment is essential for improving therapeutic strategies.

**Figure 1:**
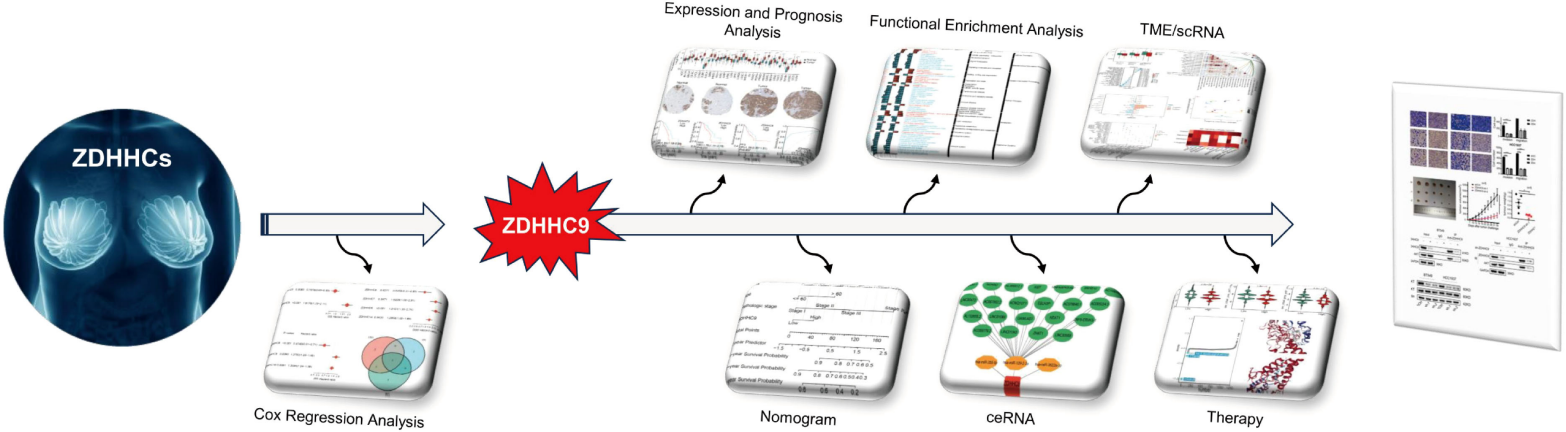
Technical route of the study.

This investigation examined the expression patterns and survival significance of 23 ZDHHC enzymes in BC, with ZDHHC9 identified as a key gene. Co-expression analysis and differential expression studies were employed to explore the possible biological functions and pathways regulated by ZDHHC9 in BC. The tumor microenvironment (TME) and single-cell data were further evaluated to understand the link between ZDHHC9 expression and immune cell infiltration. Additionally, the predictive value of ZDHHC9 for immunotherapy outcomes and drug sensitivity was assessed through various analytical approaches. This comprehensive analysis underscores ZDHHC9’s crucial function in BC, demonstrating its value as a prognostic biomarker and treatment target, thus establishing a conceptual foundation for the molecular diagnosis and clinical management of BC. The full text technical route is shown in **Figure 1**.

## 2 Methodologies and materials

### 2.1 Expression analysis

The transcriptional data from The Cancer Genome Atlas (TCGA) (https://portal.gdc.cancer.gov/) and Genotype-Tissue Expression (GTEx) (https://www.gtexportal.org/) databases were utilized to assess the differential expression of ZDHHC9 mRNA between tumor tissues and their adjacent normal counterparts. This analysis was conducted using the Xiantao tool (https://www.xiantao.love/). Additionally, the protein expression of ZDHHC9 in BC was evaluated using immunohistochemistry data obtained from the Human Protein Atlas (HPA) database (https://www.proteinatlas.org/). Statistical analyses and visualizations of ZDHHC9 expression across diverse clinicopathological characteristics in BC were performed using the Xiantao tool in combination with the “stats”, “car”, and “ggplot2” R packages.

### 2.2 Prognostic analysis

Cox regression analysis was performed on the TCGA dataset utilizing the “survival” and “forestplot” R packages to investigate the association between 23 ZDHHC enzymes and BC prognosis, encompassing overall survival (OS), disease-specific survival (DSS), and progression-free interval (PFI). Log-rank p-values and hazard ratios (HR) with 95% confidence intervals (95% CI) were calculated utilizing the “survival” package, and the outcomes were depicted through the “forestplot” package. Additionally, Kaplan-Meier analysis was applied to further assess the correlation between ZDHHC9 mRNA expression and BC OS, DSS, and PFI.

### 2.3 Construction of a nomogram

Univariate and multivariate Cox regression analyses were executed to assess the prognostic significance of ZDHHC9 and clinicopathological parameters using the TCGA-BC dataset. Independent prognostic factors were incorporated into a nomogram designed to predict the 3-, 5-, and 7-year survival of individuals with BC. The predictive accuracy of the nomogram was examined through calibration curves.

### 2.4 Pathway enrichment analysis

To investigate the potential BFs and molecular pathways of ZDHHC9 in BC, co-expression analysis was initially executed utilizing the LinkedOmics database (www.linkedomics.org/login.php) (11). The “HiSeq RNA” platform and the “TCGA_BC” cohort were chosen for this analysis, with Pearson’s correlation employed to determine associations between ZDHHC9 and co-expressed genes. Differentially expressed genes (DEGs) between high- and low-ZDHHC9 expression cohorts were identified through the “DESeq2” R package, using |log2^FC^| ≥ 1 and FDR < 0.05 as selection thresholds. The DEGs were further examined through Gene Ontology (GO) (12) and Kyoto Encyclopedia of Genes and Genomes (KEGG) pathway (13) enrichment utilizing the “clusterProfiler”, “DOSE”, and “enrichplot” R packages. A p-value of <0.05 was deemed statistically significant. Furthermore, gene set enrichment analysis (GSEA) was conducted to compare low- and high-ZDHHC9 expression cohorts, incorporating gene sets from c2.cp.reactome, c2.cp.wikipathways, c5.go.bp, c5.go.cc, and c5.go.mf using the “clusterProfiler” and “enrichplot” R packages (14).

### 2.5 LncRNA-miRNA-ZDHHC9 regulatory network analysis

Upstream miRNAs interacting with ZDHHC9 were forecasted through four online databases: miRWalk (http://mirwalk.umm.uni-heidelberg.de/), miRDB (http://mirdb.org/), miRabel (http://bioinfo.univ-rouen.fr/mirabel/index.php?page=help), and TargetScan (http://www.targetscan.org/vert_72/). Only miRNAs identified across all four databases were selected for further investigation. Candidate miRNAs with a TargetScan score ≥ 96 were prioritized for subsequent examinations. The Xiantao platform was employed to evaluate these candidate miRNA expression patterns in BC, and those exhibiting significant expression differences were retained for further evaluation. The lncRNAs targeting these miRNAs were identified via the TargetScan database. Lastly, the lncRNA-miRNA-ZDHHC9 regulatory network was generated and depicted with Cytoscape software.

### 2.6 Tumor microenvironment analysis

The ESTIMATE algorithm (15), implemented via the “estimate” R package, was utilized to infer immune cell infiltration levels (ImmuneScore) and stromal component abundance (StromalScore) in tumor samples based on gene expression data. The association, between ZDHHC9 mRNA expression and 22 tumor immune cells, was examined by “CIBERSORT” algorithm and visualized the results with “ggplot2” R package (16). Furthermore, given the important role of the tumor immune cycle in the anti-tumor immune response (17), we use the tumor immunophenotype (TIP; http://biocc.hrbmu.edu.cn/TIP/) analysis tools, to systematically investigate the potential connection between ZDHHC9 expression and key components of this immunoregulatory process.

### 2.7 Single-cell RNA sequencing analysis

The BC_EMTAB8107 single-cell dataset was procured from the ArrayExpress database (https://www.ebi.ac.uk/biostudies/arrayexpress). The “Seurat” R package was utilized to conduct quality control analysis of the scRNA-seq information, and quality control measures were implemented as follows: elimination of genes detected in <3 cells, removal of cells with <200 or >10,000 expressed genes, and exclusion of cells with >20% mitochondrial gene content. Post-quality control, gene expression data were normalized (NormalizeData) and scaled (ScaleData), with the 2,000 most variable genes selected via FindVariableFeatures for subsequent dimensional reduction. uniform manifold approximation and projection (UMAP) visualization facilitated cell cluster identification, while differential expression analysis using FindAllMarkers enabled marker-based cell typing. Cell-cell interaction patterns were subsequently modeled through CellChat’s ligand-receptor database (18).

### 2.8 Immunotherapy prediction analysis

To assess ZDHHC9 expression’s impact on immunotherapy response prediction, the EaSIeR (19) prediction model, incorporating CYT, TLS, IFN-γ, and T cell_inflamed features, was applied. Statistical differences in CYT, TLS, IFN-γ, and T cell_inflamed scores between high- and low-ZDHHC9 expression cohorts were compared utilizing Wilcoxon Rank Sum and Signed Rank Tests. Individuals with BC were stratified into four cohorts (Q1 to Q4) grounded in ZDHHC9 expression levels, with Q1 comprising the top 25% exhibiting the highest expression and Q4 representing the bottom 25% with the lowest expression. The average score of each feature was computed for every cohort, and the outcomes were depicted utilizing the “pheatmap” R package. Immunotherapy response scores for PD-1 and CTLA4 blockade (20) were extracted from the TCIA database (http://tcia.at/) to evaluate ZDHHC9’s prediction of immunotherapy outcomes. In addition, GSE91061 and phs000452, sourced from the Gene Expression Omnibus (GEO) (https://www.ncbi.nlm.nih.gov/geo/) public database, were used to evaluate the effect of immunotherapy.

### 2.9 Drug sensitivity analysis

The Connectivity Map (CMap) database (21) was employed to identify potential drug candidates grounded in specific gene expression profiles. The top 150 upregulated and downregulated DEGs from high- and low-ZDHHC9 expression cohorts were entered into the CMap database to predict small-molecule drugs for cancer treatment. Compounds with negative scores were recognized as candidates capable of reversing undesirable biological characteristics of BC, suggesting greater therapeutic potential.

### 2.10 Cell culture

The normal human breast tissue cell line (MCF-10A) and BC cell lines (BT-549, HCC1937, MDA-MB-231, SUM159PT, and MCF-7) authenticated by STR profiling were purchased from Shanghai Fuheng Biotechnology Co., LTD (Shanghai, China). MCF-10A cells were kept in Dulbecco’s Modified Eagle Medium/F12 (DMEM/F12, Procell). BC cell lines, including BT-549, HCC1937, MDA-MB-231, SUM159PT, and MCF-7 were propagated in RPMI 1640 or DMEM (Gibco, USA) comprising 10% fetal bovine serum (FBS) (Hyclone, USA) and penicillin-streptomycin (Gibco, USA). All cell lines were kept at 37°C in a moisture-controlled environment with 5% CO_2_.

### 2.11 Lentivirus infection

A lentiviral short hairpin RNA (shRNA) vector targeting human ZDHHC9, along with a non-targeting control vector, was generated by Tsingke Biotechnology Co., Ltd (Beijing, China). Targeting the shRNA sequence of ZDHHC9: shRNA-1, GTCTGTGATGGTGGTGAGAAA; shRNA-2, GAGGAACTACCGCTACTTCTA. Plasmid transfection was executed utilizing Lipofectamine 3000 (Thermo Fisher Scientific, Inc.) per the supplier’s protocol. The viral supernatant was harvested to infect BT-549 and HCC1937 cells, and stable ZDHHC9 knockdown BC cells were selected using puromycin.

### 2.12 Western blot analysis

Cells underwent lysis utilizing RIPA buffer (Beyotime, China) comprising both protease and phosphatase inhibitors. Protein levels were quantified utilizing the BCA method, and the specimens were separated by 10% SDS-PAGE gel. Afterward, proteins were moved into PVDF membranes and subsequently blocked. These membranes underwent overnight exposure to primary antibodies (anti-ZDHHC9, Abcam, UK; anti-GAPDH, Proteintech, USA; anti-AKT, Cell Signaling Technology, USA; anti-Phospho-Akt, Cell Signaling Technology, USA; anti-β-actin, Abcam, UK) at 4°C, succeeded by exposure to HRP-linked secondary antibodies (Proteintech, USA) for 1 h. Protein bands were identified through chemiluminescence imaging.

### 2.13 Macrophage polarization detection

Conditioned media (CM) were collected from BT549 and HCC1937 cells. The CM was centrifuged (3000×g, 10 minutes) and filtered (0.22 microns) to remove cell debris. THP-1 monocytes were treated with 100 nM PMA (MedChemExpress, USA) for 48 hours to differentiate them into macrophages. Then, the differentiated macrophages were stimulated with 50% (v/v) CM for 24 hours. The polarization state was analyzed by flow cytometry and western blotting. The required markers for the experiment were as follows: CD86-APC (Invitrogen, USA) and CD206-FITC (Invitrogen, USA). The primary antibodies were as follows: anti-Arg1 (Thermo Fisher, USA), anti-CD206 (Abcam, UK), anti-iNOS (Abcam, UK), anti-CD86 (Abcam, UK).

### 2.14 Co-immunoprecipitation

Protein interactions were analyzed using an Immunoprecipitation Kit (Abcam, ab206996). Briefly, cell lysates were prepared to release total proteins, followed by pre-clearing with Protein A/G beads to reduce non-specific background. Specific primary antibodies were incubated with the pre-cleared lysates at 4°C overnight with gentle rotation. Protein A/G beads were then added to capture antibody-target protein complexes, and the beads were isolated via low-speed centrifugation (3,000 × g, 5 min). Subsequent washes (3×) with ice-cold IP buffer removed unbound proteins and contaminants. Target proteins and their interactors were eluted using either low-pH elution buffer or heating (95°C, 5 min) in 1× Laemmli buffer. Eluted proteins were finally resolved by Western Blot analysis.

### 2.15 CCK-8 assay

BC cells were placed in 96-well plates at 1500 cells/mL. Cell viability was evaluated at 24, 48, 72, 96, and 120 h. Following the addition of 10 μL of CCK-8 reagent to individual wells and incubating for 1 h, absorbance at 450 nm was recorded utilizing a microplate reader (Thermo, USA).

### 2.16 Colony formation assay

Approximately 1000 control or ZDHHC9 knockdown BT-549 and HCC1937 cells were placed in 6-well plates and maintained at 37°C in a 5% CO_2_ atmosphere for two weeks. The cells were subsequently fixed utilizing 10% methanol and stained with 0.1% crystal violet.

### 2.17 Transwell assay

5 × 10^4^ BT-549 and HCC1937 cells were used for cell migration, while 1 × 10^5^ BT-549 and HCC1937 cells were used for cell invasion. These cells were placed in the upper chambers of transwell inserts (Merck Millipore, USA) with serum-free medium, while the lower chambers were supplied with a medium comprising 10% FBS. Following one day of incubation, cells were stabilized utilizing 5% paraformaldehyde and stained with crystal violet dye (Beyotime, China). The specimens were documented through a light microscope (Olympus, Japan).

### 2.18 IC_50_ determination

Cell viability was assessed via CCK-8 assay after 48 hours Imatinib (Selleckchem, USA) treatment. Concentration gradients were:BT549: 0, 1, 2, 4, 8, 16 μM;HCC1937: 0, 1, 4, 8, 16, 32 μM. Dose-response curves were generated in GraphPad Prism 9.0.

### 2.19 Flow cytometry

BT-549 and HCC1937 cells transfected with control or shRNA were inoculated into 6-well plates at a density of 2 × 10^5^ cells per well, cultured under standard conditions for 48 h, and then subjected to trypsin isolation and PBS washing. Subsequently, the cells were exposed to Annexin V and propyl iodide (PI) and analyzed by flow cytometry. The apoptosis of BC cells was detected using FlowJo software (FlowJo, LLC, Bethesda, USA).

### 2.20 Immunohistochemistry

Human BC specimens (n=20) were collected from Shenzhen Hospital of Southern Medical University. IHC was performed on FFPE sections using: Primary antibodies: ZDHHC9 (Abcam, UK), CD68 (Abcam, UK), CD86(Abcam, UK), CD206(Abcam, UK). Staining protocol: Antigen retrieval (EDTA pH 9.0, 100°C, 20 min) → Primary antibody incubation (4°C, overnight) → DAB visualization → Hematoxylin counterstaining. Quantitative analysis: Integrated Optical Density (IOD) of positive staining was calculated using ImageJ (NIH) with threshold OD > 0.2.

### 2.21 Xenograft experiments

Four to five-week-old BALB/c nude mice were accommodated under stable environmental conditions. The experimental protocols received approval from the Animal Ethics Committee of Shenzhen Hospital of Southern Medical University, and strictly adhered to the Guide for the Care and Use of Laboratory Animals. The mice were arbitrarily allocated into three experimental cohorts, each consisting of five mice. To generate BT-549 xenografts, either 2×10^6^ BT-549 cells or 2×10^6^ BT-549 cells with ZDHHC9 knockdown were injected subcutaneously into the mice. Tumor growth was tracked over the course of five weeks, with measurements recorded approximately every three days. After the study concluded, the mice were sacrificed, after which the tumors were excised, weighed, and their volumes calculated.

### 2.22 Acyl-biotin exchange assay

Palmitoylation of AKT protein was analyzed using the Acyl-Biotin Exchange (ABE) assay according to established protocols (22). Briefly, total cellular proteins were extracted and treated with 25 mM N-ethylmaleimide (NEM) at 4°C for 1 h with vigorous shaking (1200 rpm) to block free thiol groups. Target proteins were immunoprecipitated using anti-AKT antibody (Cell Signaling Technology, USA) conjugated to protein A/G magnetic beads (Thermo Fisher, USA). Purified protein complexes were then incubated with 1 M hydroxylamine (HAM, pH 7.2) at room temperature for 1 h to cleave palmitoyl-thioester bonds. Subsequently, samples were reacted with 5 μM Biotin-BMCC (Thermo Fisher, USA) at 4°C for 2 h for biotinylation of newly exposed thiols. Bead-bound complexes were resuspended in 40 μL 2× Laemmli SDS sample buffer, denatured at 100°C for 10 min, and resolved by SDS-PAGE. Biotin-labeled proteins were detected by Western blot using streptavidin-HRP (1:5000; Abcam, UK).

### 2.23 Statistical analysis

Bioinformatics analysis was executed utilizing R software (version 4.1.2). The Student’s t-test facilitated comparisons between two cohorts, while one-way non-parametric ANOVA was employed for comparisons involving three or more cohorts. Statistical analyses were carried out utilizing GraphPad Prism (version 8.4.0). The p value was corrected by the Benjamini-Hochberg method. A p-value below 0.05 was deemed statistical significance. (ns, p≥0.05, *p < 0.05, **p < 0.01, ***p < 0.001).

## 3 Results

### 3.1 ZDHHC9 is closely associated with BC

**Figure 2:**
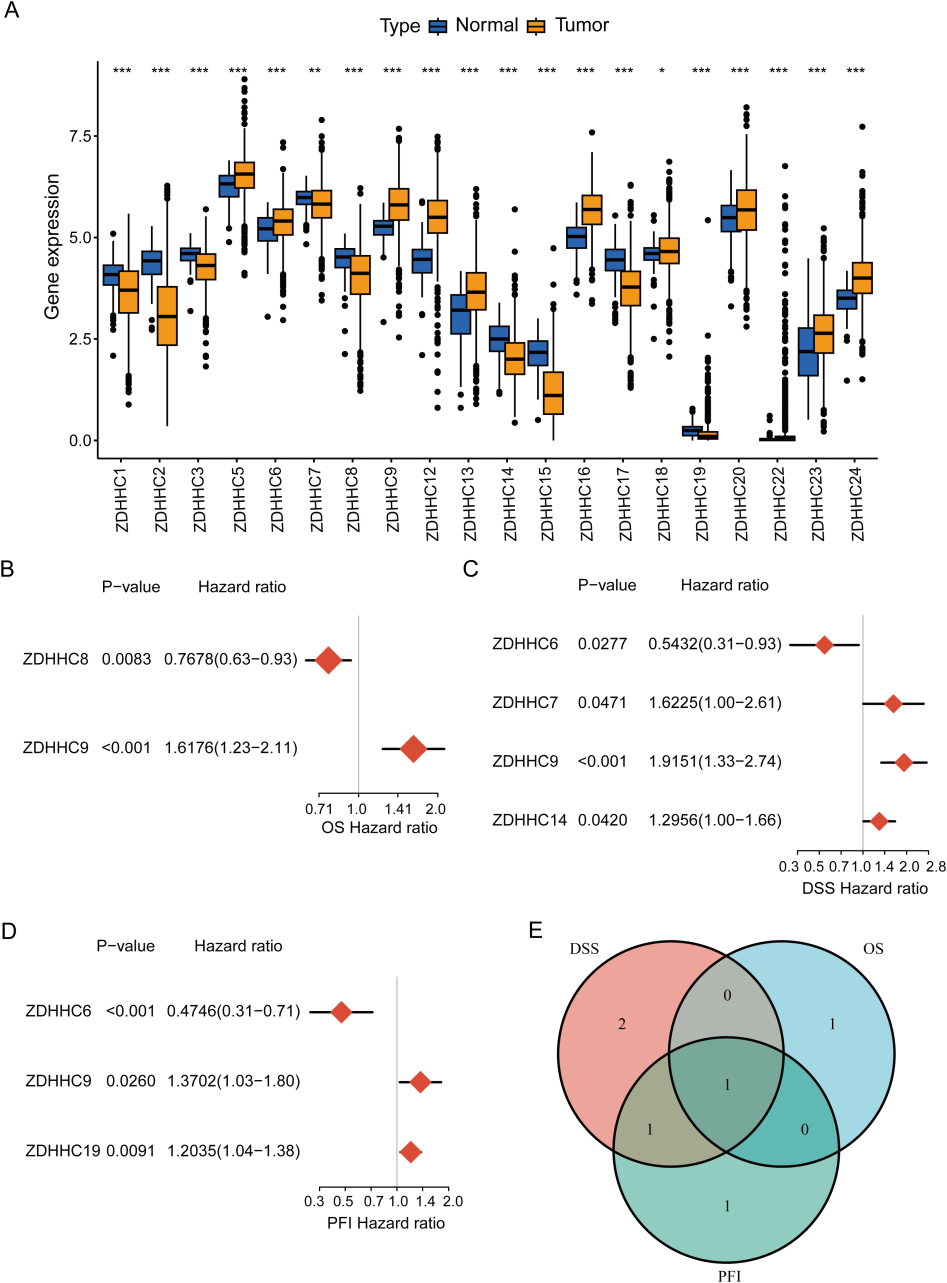
Determination of ZDHHC9. **(A)** Differential expression of 23 ZDHHC genes between normal and BC samples. The asterisks indicate statistical significance. **(B–D)** Forest plots showing the correlation between ZDHHC expression and OS, DSS, or PFI in BC. A HR > 1 denotes the gene is a risk factor, while HR < 1 denotes a protective factor. **(E)** Venn diagram shows the intersection of OS, DSS, and PFI for the ZDHHC genes. (*p < 0.05, **p < 0.01, ***p < 0.001).

To explore the ZDHHC enzymes most relevant to BC, expression levels of 23 ZDHHCs in BC were initially analyzed. The findings indicated that 20 ZDHHCs exhibited abnormal expression, with 11 genes being upregulated and 9 downregulated (P < 0.05) (**Figure 2A**). Additionally, univariate Cox regression analyses were employed to evaluate the overall prognostic (including OS, DSS, and PFI) relevance of ZDHHCs in BC. As illustrated in **Figures 2B–D**, ZDHHC9 was linked to elevated OS mortality risk (HR > 1 and P < 0.001), ZDHHC7, ZDHHC9, and ZDHHC14 were associated with higher DSS mortality risk (HR > 1 and P < 0.05), ZDHHC9, and ZDHHC19 correlated with increased PFI mortality risk (HR > 1 and P < 0.05). The Venn diagram demonstrated that ZDHHC9 was a shared risk factor across all three survival analyses (**Figure 2E**). These observations indicate that among the 23 ZDHHCs, ZDHHC9 serves a pivotal function in BC.

### 3.2 ZDHHC9 is elevated in BC and associated with unfavorable outcomes

**Figure 3:**
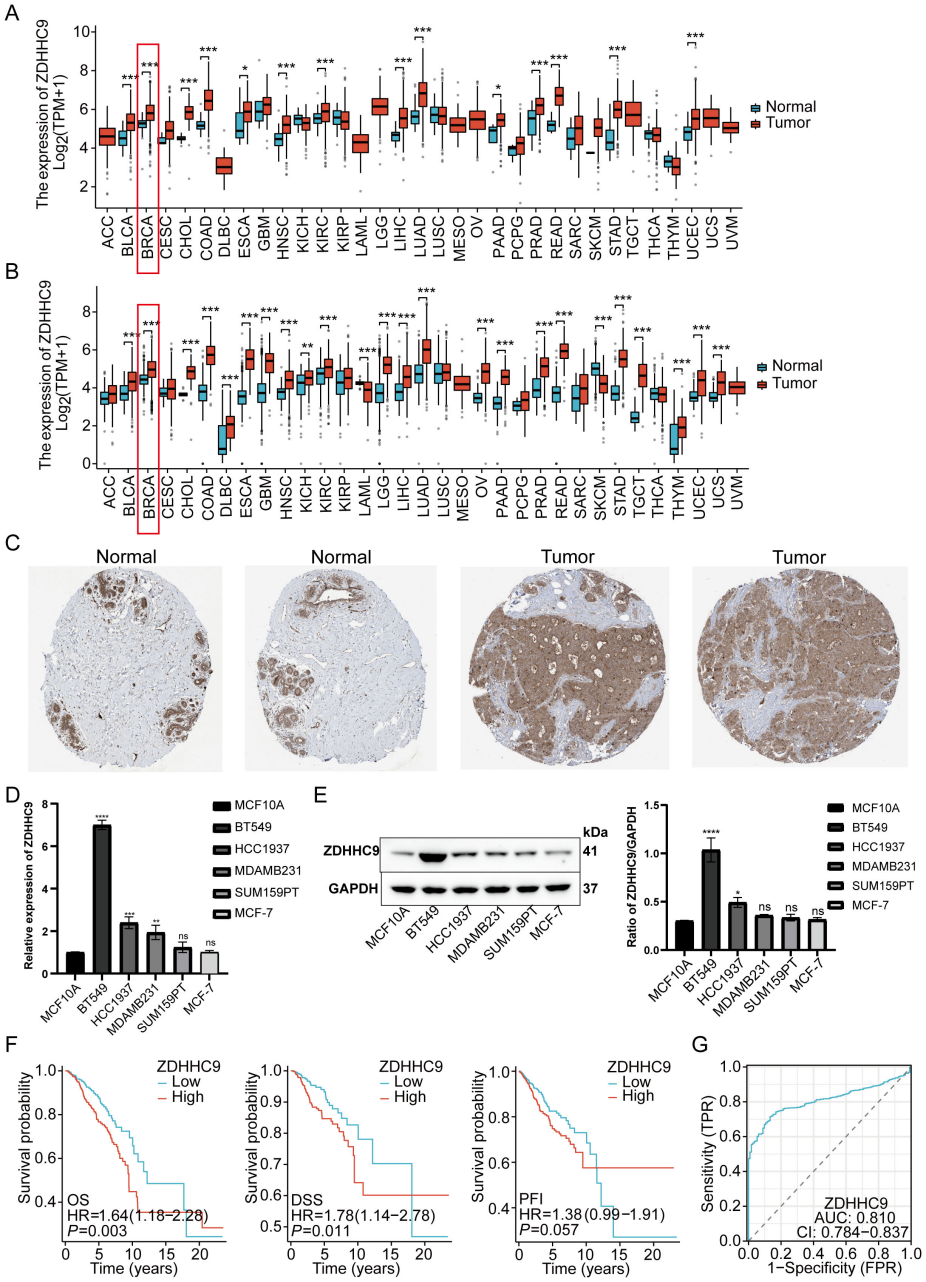
Tissue expression and prognosis analysis of ZDHHC9. **(A)** ZDHHC9 expression levels in various cancers based on TCGA data. **(B)** ZDHHC9 expression levels in various cancers combining TCGA and GTEx databases. **(C)** Immunohistochemistry analysis of ZDHHC9 protein expression in normal breast tissues and BC tissues. **(D)** RT-qPCR measured ZDHHC9 mRNA expression in normal breast cells and five BC cell lines. **(E)** Western blot assessed ZDHHC9 protein expression in normal breast cells and BC cell lines. **(F)** Survival curves showing OS, DSS, and PFI for BC patients with low or high ZDHHC9 expression. **(G)** ROC curve analysis of the diagnostic value of ZDHHC9. (*p < 0.05, **p < 0.01, ***p < 0.001).

To evaluate the impact of ZDHHC9 on BC, a comprehensive analysis was performed utilizing multiple databases. Initially, ZDHHC9 mRNA expression levels were examined across 33 cancers through the TCGA and GTEx databases. The findings suggested that ZDHHC9 mRNA expression was elevated in most tumor tissues (P < 0.05), including BC, relative to corresponding normal tissues, both within the TCGA dataset independently and in the combined TCGA-GTEx dataset (**Figures 3A, B**). Additionally, immunohistochemistry analysis sourced from the HPA database corroborated the abnormal increase in ZDHHC9 protein levels in BC patient samples (**Figure 3C**). To further verify this result, ZDHHC9 expression was examined at both mRNA and protein levels across five BC cell lines. The outcomes indicated that both mRNA (**Figure 3D**) and protein (**Figure 3E**) levels of ZDHHC9 were elevated in BT-549 and HCC1937 cells relative to those observed in normal breast cells (MCF-10A) (**Figures 3D, E**). In addition, Kaplan-Meier survival analysis uncovered that the individuals with BC exhibiting reduced ZDHHC9 expression demonstrated superior OS, DSS, and PFI outcomes despite the non-significant p-value for PFI (**Figure 3F**). Furthermore, ROC analysis demonstrated that ZDHHC9 possessed significant diagnostic potential in BC, reflected by an AUC value of 0.81 (**Figure 3G**). These results underscore that ZDHHC9 is markedly overexpressed in BC and linked to unfavorable clinical outcomes, necessitating further exploration.

### 3.3 Construction of a ZDHHC9-based nomogram

**Figure 4:**
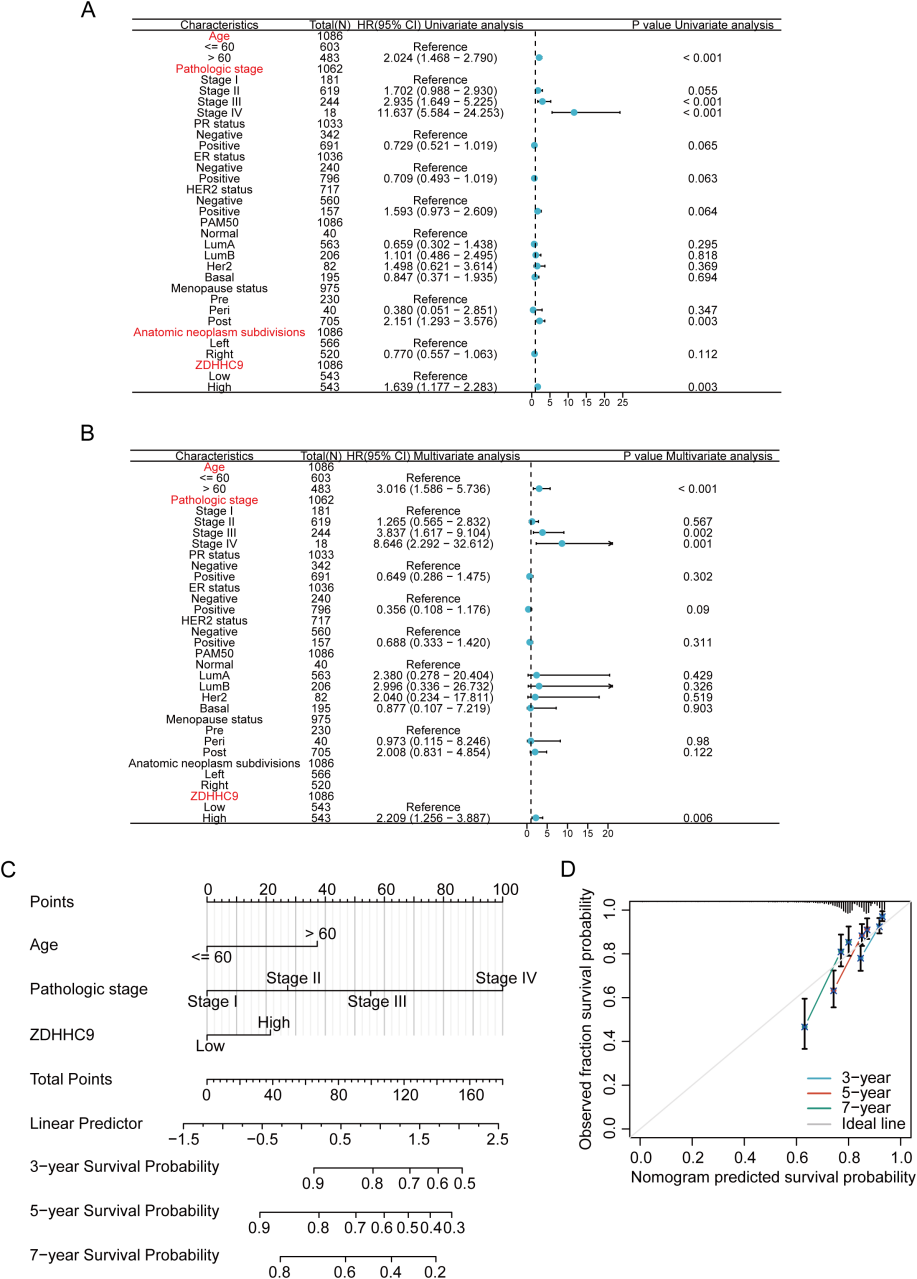
Construction of nomogram. **(A, B)** Univariate **(A)** and multivariate **(B)** analyses of ZDHHC9 and various clinicopathological parameters in BC. **(C)** Construction of nomogram by clinicopathological parameters and ZDHHC9 in the TCGA-BC cohort. **(D)** Calibration curves for the nomogram predicting 3-, 5-, and 7-year OS in BC patients.

Given the influence of ZDHHC9 expression on BC patient outcomes, survival outcomes were evaluated based on differing ZDHHC9 expression levels. Patients exhibiting low ZDHHC9 expression (Q3, Q4 cohort) displayed higher survival rates in comparison to those with elevated expression (Q1, Q2 cohort) (**Supplementary Figure S1A**). To accurately predict 3-, 5-, and 7-year survival probabilities, a nomogram was developed. Since multiple factors, including clinical variables like age, tumor stage, and cancer subtype, influence BC prognosis, univariate and multivariate Cox regression analyses were executed by integrating ZDHHC9 expression alongside these pathological characteristics. The results from both univariate and multivariate analyses ascertained age, stage, and ZDHHC9 expression as independent prognostic indicators associated with BC patient outcomes (P < 0.05) (**Figures 4A, B**). Consequently, a nomogram integrating ZDHHC9 expression with age and stage was formulated to predict 3-, 5-, and 7-year OS for individuals with BC (**Figure 4C**). Calibration curves demonstrated a high degree of concordance between the nomogram’s predictions and observed outcomes, affirming its reliability (**Figure 4D**).

### 3.4 Potential functional enrichment of ZDHHC9 in BC

To investigate the biological significance of ZDHHC9 in BC, co-expression networks associated with ZDHHC9 were analyzed using LinkedOmics. The analysis identified 4,860 genes exhibiting positive correlations and 7,718 genes showing negative correlations with ZDHHC9 (FDR < 0.05) (**Supplementary Figure S2A**). Heatmaps illustrated the 50 genes exhibiting maximum positive and negative correlations to ZDHHC9 (**Supplementary Figures S2B, C**). Subsequently, GSEA was utilized to determine the GO_BP terms and KEGG pathways associated with ZDHHC9 co-expressed genes. GO_BP analysis indicated that positively co-expressed genes were primarily linked to biological processes such as cell cycle activity and transcriptional regulation, while negatively co-expressed genes participated in interleukin production, cytokine-mediated processes, immune responses, and related functions (**Supplementary Figure S2D**). The KEGG pathway examination demonstrated that positively co-expressed genes were enriched in transcriptional and translational regulation pathways (encompassing protein processing in the endoplasmic reticulum, ribosome biogenesis in eukaryotes, and RNA transport) and cell cycle-related pathways (such as oocyte meiosis and homologous recombination). Conversely, genes exhibiting negative co-expression were concentrated in immune system-associated pathways, encompassing cytokine-cytokine receptor interaction, primary immunodeficiency, intestinal immune network for IgA production, and autoimmune thyroid disease (**Supplementary Figure S2E**).

**Figure 5:**
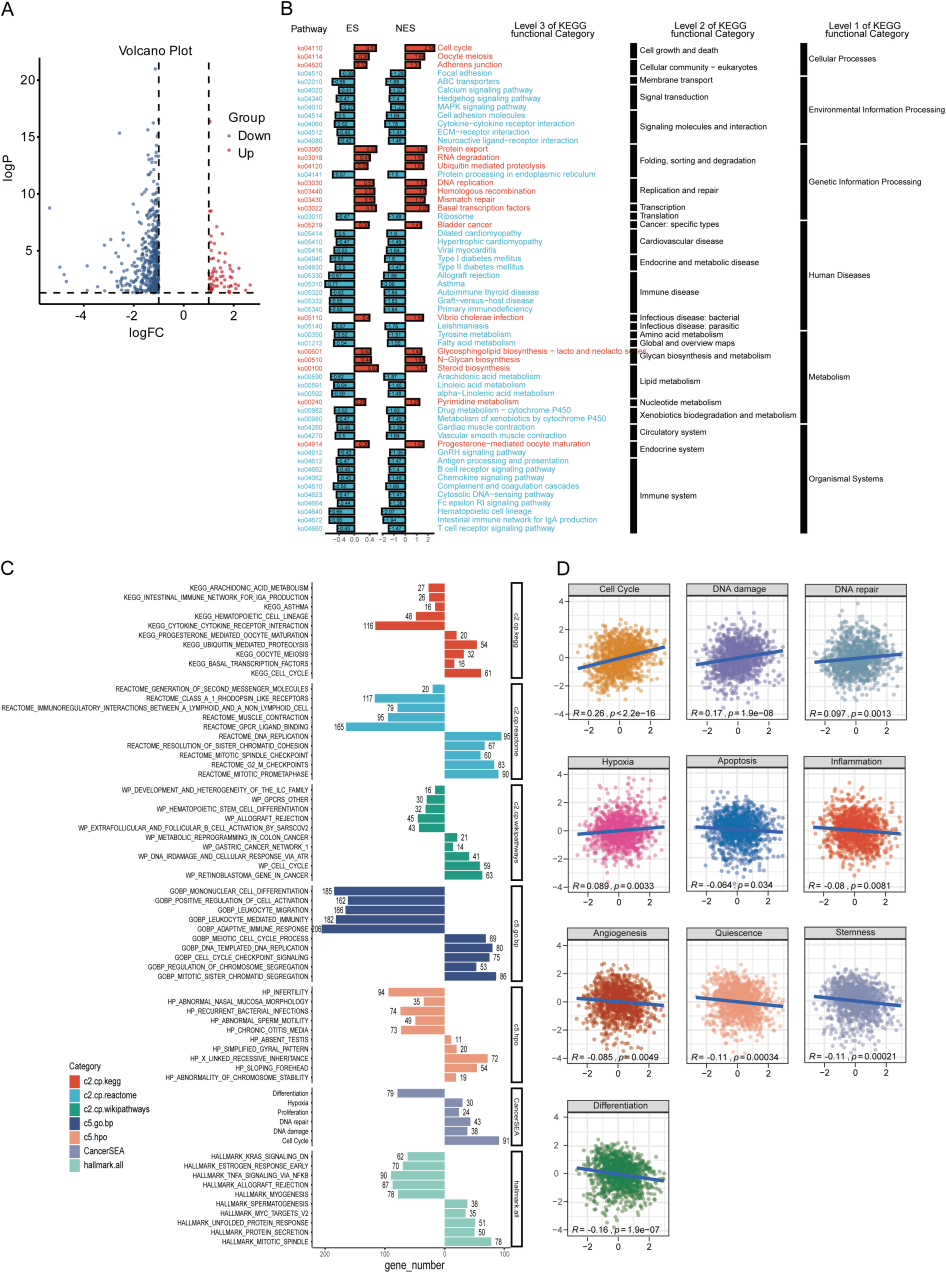
Functional analysis of ZDHHC9. **(A)** Volcano plot showing DEGs between high and low ZDHHC9 expression cohorts. **(B)** KEGG pathway enrichment analysis of DEGs. **(C)** Enrichment plots of multiple relevant pathways from GSEA for ZDHHC9. Different colors represent different gene sets. **(D)** Pearson correlation between ZDHHC9 and 14 malignant features analyzed by GSVA.

To further investigate the possible molecular mechanisms of ZDHHC9 in BC, differential gene expression analysis was conducted between high and low ZDHHC9 expression cohorts, identifying 487 DEGs, with 424 downregulated (blue) and 63 upregulated (red) genes (**Figure 5A**). KEGG enrichment analysis of these DEGs indicated that the high ZDHHC9 expression cohort was predominantly linked to oncogenic signaling pathways, including processes like cell growth and death, folding, sorting and degradation, as well as replication and repair. Conversely, the low ZDHHC9 expression cohort was enriched in pathways linked to the immune system, immune diseases, and environmental information processing (**Figure 5B**). GSEA provided further insights into the biological pathways regulated by ZDHHC9, revealing that high ZDHHC9 expression activated oncogenic pathways like the cell cycle, DNA damage, DNA repair, and chromosome regulation, while low ZDHHC9 expression stimulated immune-related pathways, including immunoregulatory interactions between a lymphoid/non-lymphoid cell interactions, adaptive immune response, extrafollicular and follicular B cell activation by SARS-CoV-2, and leukocyte-mediated immunity (**Figure 5C**). Additionally, GSVA evaluation demonstrated positive associations between ZDHHC9 expression and oncogenic functions, encompassing the cell cycle, DNA damage, DNA repair, and hypoxia (P < 0.05) (**Figure 5D**). These findings suggest that aberrant ZDHHC9 expression may promote carcinogenic behaviors in BC by influencing cell cycle regulation, DNA repair mechanisms, and tumor immunity, suggesting its value as a therapeutic target for BC treatment.

### 3.5 Effect of ZDHHC9 knockdown on BC

**Figure 6:**
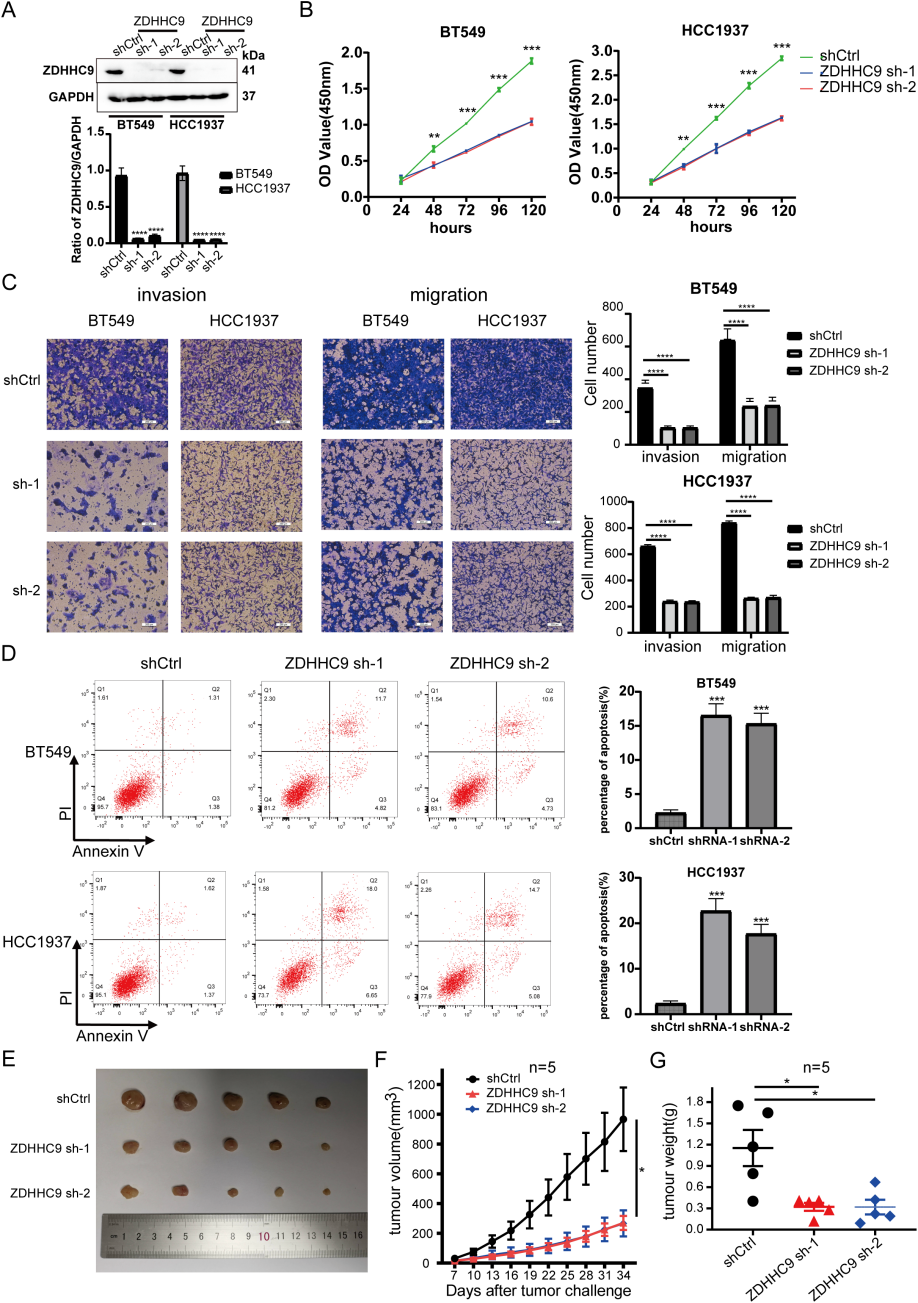
Expression of ZDHHC9 and knockdown efficiency of sh-ZDHHC9. **(A)** Western blot showed the efficacy of sh-ZDHHC9 in BT-549 and HCC1937 cells. **(B)** CCK-8 assay was used to assess the proliferation of BT-549 and HCC1937 cell lines. **(C)** Transwell assay was utilized to evaluate the invasion and migration abilities of BT-549 and HCC1937 cells. **(D)** Flow cytometry was used to detect the effect of knockdown of ZDHHC9 on cell apoptosis, and the associated quantification is displayed in a bar graph. **(E)** Tumors derived from both shCtrl and sh-ZDHHC9 cohorts. **(F, G)** Tumor volume **(F)** and weight **(G)** were measured in the shCtrl or sh-ZDHHC9 cohorts. (*p < 0.05, **p < 0.01, ***p < 0.001).

Considering that the high expression of ZDHHC9 was related to the biological behavior of BC, shRNA was utilized to knock down ZDHHC9 expression in BT-549 and HCC1937 cells. The knockdown efficiency was confirmed through RT-qPCR (**Supplementary Figure S3A**) and WB (**Figure 6A**). A subsequent CCK-8 assay was performed to evaluate the proliferation of BC cells. The results revealed that ZDHHC9 knockdown markedly suppressed the proliferation of BT-549 and HCC1937 cells (**Figure 6B**). This inhibitory effect was further corroborated by a colony formation assay (**Supplementary Figure S3B**). Subsequently, transwell assay indicated that ZDHHC9 silencing substantially impaired the invasive and migratory capabilities of BC cells (**Figure 6C**). Moreover, apoptosis experiments were performed, and found that ZDHHC9 knockdown markedly promoted apoptosis of tumor cells (**Figure 6D**). Additionally, given that high ZDHHC9 expression correlates with unfavorable prognosis in BC patients, nude mice were subcutaneously injected with either ZDHHC9 knockdown or control tumor cells. Tumors derived from the ZDHHC9 knockdown cohort exhibited a marked reduction in size (**Figure 6E**), volume (**Figure 6F**), and weight (**Figure 6G**) compared to those in the control cohort (**Figures 6E–G**). In summary, these observations collectively underscore ZDHHC9’s essential function in promoting tumorigenesis and progression in BC.

### 3.6 Construction of the upstream ceRNA regulatory network for ZDHHC9

**Figure 7:**
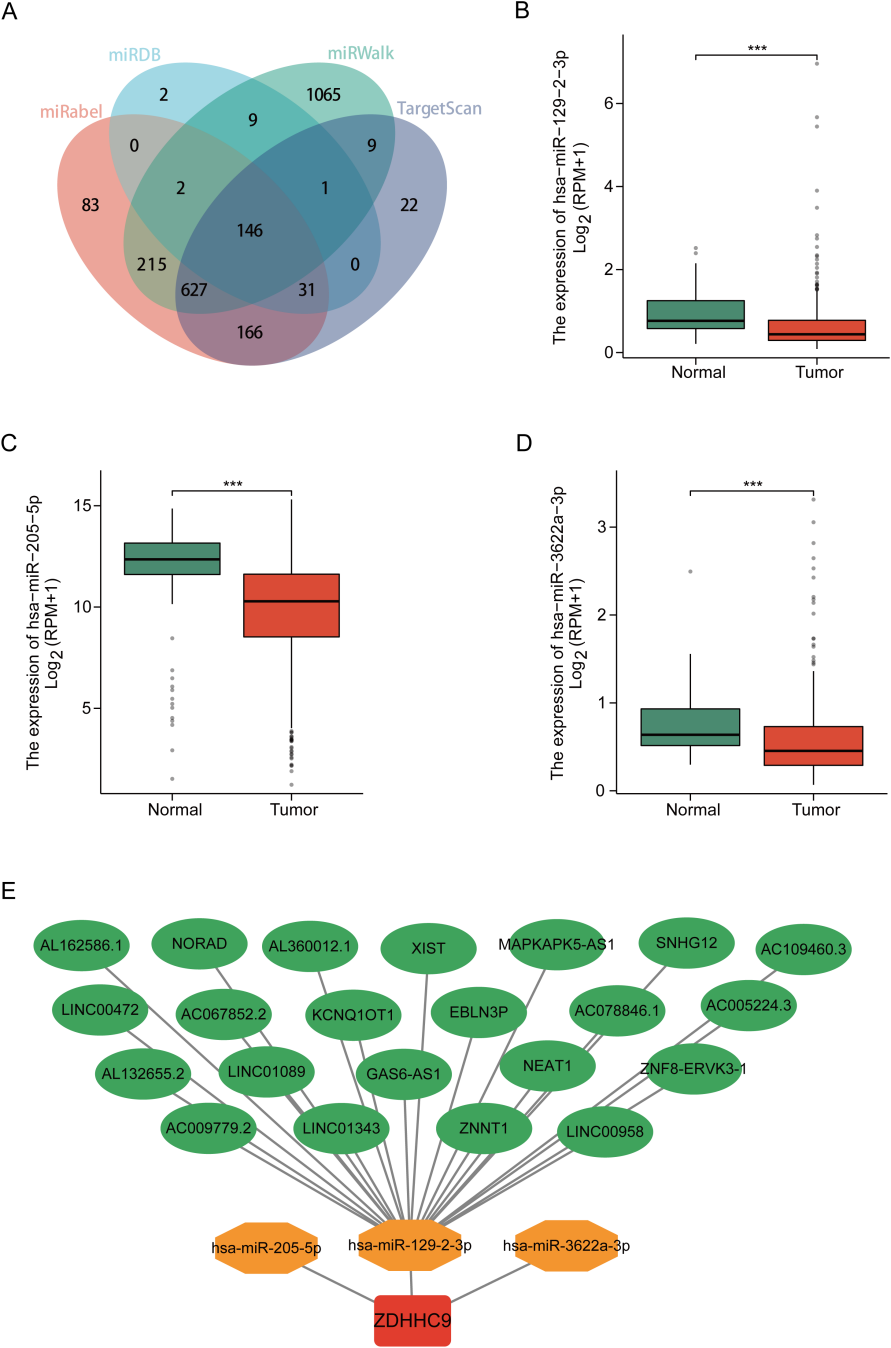
Construction of the ceRNA network. **(A)** Venn diagram displaying miRNAs targeting ZDHHC9 predicted by miRabel, miRDB, miRWalk, and TargetScan. **(B–D)** Expression levels of miRNAs in BC. **(E)** The ceRNA network constructed by Cytoscape, including ZDHHC9, three miRNAs, and 22 lncRNAs. (***p < 0.001).

Studies have shown that long non-coding RNAs (lncRNAs) serve an essential function in tumor progression by modulating downstream mRNA expression through the sequestration of target miRNAs (23). In this study, the potential lncRNA-miRNA regulatory network involved in the regulation of ZDHHC9 expression was explored. Initially, the miRWalk, miRDB, TargetScan, and miRabel databases were screened, resulting in the identification of 146 candidate miRNAs targeting ZDHHC9 mRNA (**Figure 7A**). Further analysis with TargetScan yielded 48 miRNAs with a score ≥96. Bioinformatics analysis revealed that only hsa-miR-129-2-3p (**Figure 7B**), hsa-miR-205-5p (**Figure 7C**), and hsa-miR-3622a-3p (**Figure 7D**) exhibited negative correlations with ZDHHC9 in BC (P < 0.05) (**Figures 7B–D**). Additionally, 22 lncRNAs targeting hsa-miR-129-2-3p were identified through TargetScan; however, no lncRNAs were found to target hsa-miR-205-5p or hsa-miR-3622a-3p. Finally, the lncRNA-miRNA-ZDHHC9 regulatory network was developed utilizing Cytoscape (**Figure 7E**). These findings suggest that the upstream lncRNA-miRNA interactions could modulate the irregular expression patterns of ZDHHC9 in BC.

### 3.7 Immune landscape of ZDHHC9 in BC

**Figure 8:**
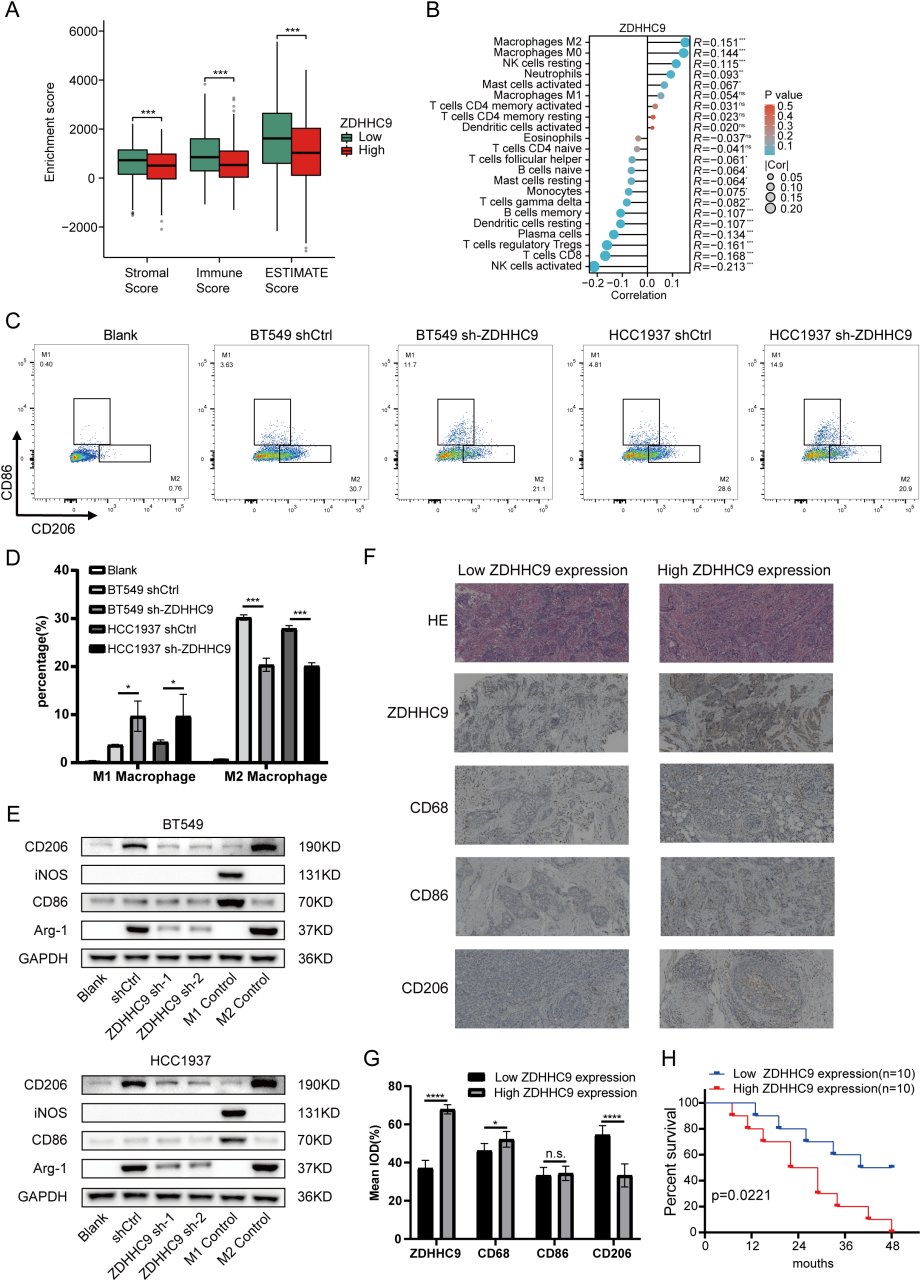
Immune landscape of ZDHHC9. **(A)** Estimate algorithm evaluated the TME scores between high- and low-ZDHHC9 expression cohorts. **(B)** Cibersort algorithm analyzed the correlation between ZDHHC9 expression and 22 tumor-infiltrating immune cells. **(C, D)** Flow cytometry analysis **(C)** and related quantitative bar charts **(D)** were used to analyze the polarization of macrophages. Conditioned media (CM) from control (shCtrl) or ZDHHC9-knockdown (sh-ZDHHC9) BT549 and HCC1937 cells were applied to PMA-differentiated THP-1 macrophages. “Blank” refers to the THP-1 cells that have undergone PMA differentiation but have not received CM stimulation. **(E)** Western blotting analysis of macrophage polarization markers. Conditioned media (CM) from control (shCtrl) or ZDHHC9-knockdown (ZDHHC9 sh-1 and ZDHHC9 sh-2) BT549/HCC1937 cells were applied to PMA-differentiated THP-1 macrophages. “Blank” refers to the THP-1 cells that have undergone PMA differentiation but have not received CM stimulation. “M1 Control” refers to the situation where THP-1 is induced to differentiate by PMA and then treated with LPS (100 ng/mL) + IFN-γ (20 ng/mL) for 48 hours, causing it to polarize towards the M1 state. “M2 Control” refers to the scenario where THP-1 is induced to differentiate by PMA and then treated with IL-4 (20 ng/mL) for 48 hours, leading it to polarize towards the M2 state. **(F)** HE staining and IHC analysis of high and low ZDHHC9 expression groups in BC tissues. **(G)** IHC analysis was conducted to determine the expression levels of ZDHHC9, CD68, CD86, and CD206 in human BC tissue samples (n = 20). The results were quantitatively analyzed using Image J.ZDHHC9 expression groups were dichotomized based on the median IOD value (53.12) from 10 BC samples: low expression (IOD < 53.12, n=10) and high expression (IOD ≥ 53.12, n=10). **(H)** Kaplan–Meier plot of survival time in BC patients with low and high ZDHHC9 expression. (ns P ≥ 0.05, * P < 0.05, *** P < 0.001, **** P < 0.0001).

Previous research has demonstrated that tumor-infiltrating immune cells (TIICs) represent a vital element of the TME and serve a function in tumor initiation, progression, and metastasis (24). To explore the link between ZDHHC9 expression and TIICs, the associations between ZDHHC9 expression and ESTIMATE scores in BC were firstly analyzed. The results suggested that ZDHHC9 expression was negatively associated to immune, stromal, and total ESTIMATE scores (P < 0.001) (**Figure 8A**), suggesting that higher ZDHHC9 expression is linked to reduced immune and stromal infiltration, resulting in increased tumor purity. Subsequently, the link between ZDHHC9 expression and immune cell infiltration in BC was examined utilizing the cibersort algorithm with spearman correlation analysis. As depicted in **Figure 8B**, elevated ZDHHC9 expression was positively linked to the infiltration of M2, M0, and resting NK cells (P < 0.05). Based on this, we verified this finding. After cell transfection with shRNA-ZDHHC9 and shCtrl, the supernatants of BT549 and HCC1937 cells were co-cultured with THP-1 cells. CD206 is a biomarker for the polarization of macrophages to M2. Flow cytometry indicated that after BC cells knocked down ZDHHC9, the proportion of M2-polarized macrophages decreased (P < 0.05), that is, its culture supernatant could inhibit the polarization of macrophages to M2 (**Figures 8C, D**). This result was also verified in the subsequent western blotting analysis (**Figure 8E**). In addition, IHC experiments revealed that in human BC tissues with high expression of ZDHHC9, the proportion of macrophage (CD68) infiltration was higher than that in the tissues with low expression of ZDHHC9, and in BC tissues with low expression of ZDHHC9, macrophages were more inclined to polarize towards M2 (CD206) (**Figures 8F, G**). Furthermore, survival analysis revealed that patients with lower expression of ZDHHC9 had a better prognosis (**Figure 8H**). Moreover, immune modulators serve an essential function in shaping the TME and influencing the effectiveness of cancer immunotherapy (25, 26). To explore ZDHHC9’s involvement in immunotherapy, correlations between its expression and various immune modulators (chemokines, immunoinhibitors, immunostimulators, and human leukocyte antigens) were analyzed. The analysis demonstrated notable associations between ZDHHC9 expression and several immune modulators. Notably, ZDHHC9 expression was negatively correlated with the immunoinhibitory genes PD-1 (PDCD1) and CTLA4 (P < 0.05), although no statistical significance was observed with PD-L1 (CD274) (**Supplementary Figure S4A**). In addition, the cancer-immunity cycle plays a critical role in antitumor immune responses (17). As depicted in **Supplementary Figure S4B**, ZDHHC9 expression displayed a negative association with the tumor immune cycle, particularly in step 5, where immune cell infiltration into tumor tissues was notably suppressed. These findings suggest that ZDHHC9 expression may alter the tumor immune microenvironment (TIME) in BC, transforming it from an immune-active state to an immune-inhibited one, thereby impacting cancer prognosis and responses to immunotherapy.

### 3.8 Single-cell analysis of ZDHHC9 in BC

**Figure 9:**
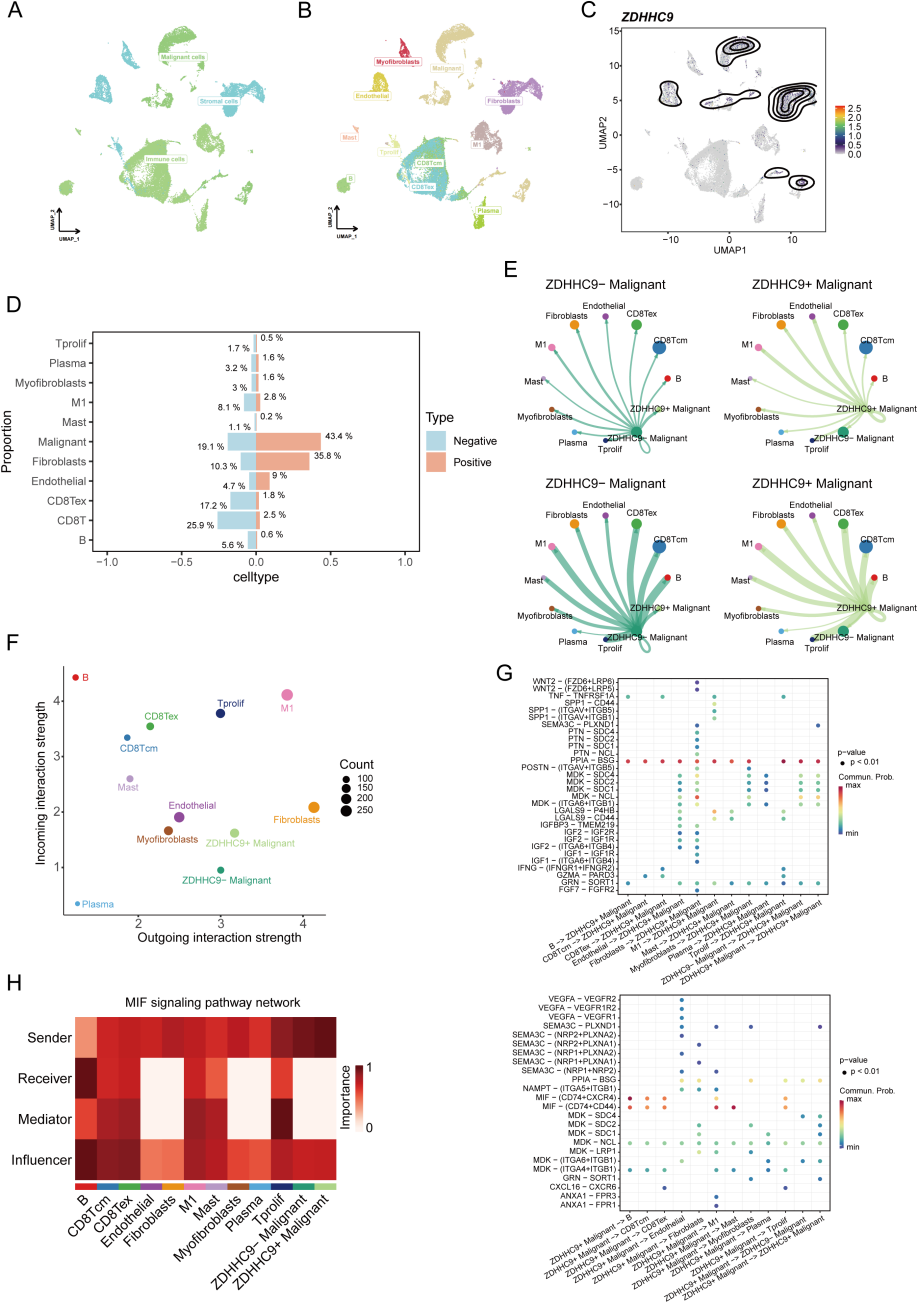
Single-cell data analysis of ZDHHC9. **(A, B)** UMAP plots visualizing cell types’ distribution and dissimilarity in EMTAB8107 data. **(C)** UMAP plot showing ZDHHC9 expression. **(D)** Bar chart displaying the proportion of different cell types in ZDHHC9-positive versus ZDHHC9-negative cohorts. **(E)** Analysis of the number of interactions and interaction strength among different cell types in ZDHHC9+ versus ZDHHC9− malignant samples using CellChat. **(F)** Scatter plot showing the difference of cell types between outgoing interactions and incoming interaction strength. **(G)** Identification of signaling by comparing the communication probabilities mediated by ligand–receptor pairs from ZDHHC9+ Malignant cells to other cell types. **(H)** Heatmap showing ligand-receptor interactions of ZDHHC9+ malignant cells with other cell types in the MIF signaling pathway.

After performing quality control and applying UMAP dimensionality reduction to the EMTAB8107 dataset, BC cells were classified into three major types (**Figure 9A**) and further divided into 11 detailed subtypes (**Figure 9B**). The UMAP plot revealed that ZDHHC9 expression was predominantly observed in malignant cells and fibroblasts (**Figure 9C**). A bar chart illustrated that the proportion of malignant cells and fibroblasts in the high-ZDHHC9 expression cohort was substantially greater than that in the low-expression cohort, whereas the proportions of CD8^+^ T cells, CD8^+^ Tex cells, and M1 macrophages were higher in the low-ZDHHC9 expression cohort (**Figure 9D**). To investigate the role of ZDHHC9-expressing malignant cells within the BC TME, the CellChat tool was employed to analyze their communication with the other 10 cell types. The analysis indicated that ZDHHC9+ malignant cells exhibited more interactions with M1 macrophages than their ZDHHC9 negative counterparts (**Figure 9E**). Furthermore, ZDHHC9+ malignant cells demonstrated stronger outgoing signal strength, while M1 macrophages displayed higher incoming signal strength (**Figure 9F**). Additional investigation revealed that the MIF-(CD74+CD44) signaling pathway mediated the most robust interaction between ZDHHC9+ malignant cells (acting as Senders) and M1 macrophages (serving as Mediators) (**Figures 9G, H**), suggesting that BC TME could be influenced through MIF-(CD74+CD44) signal transduction. These findings suggest that aberrant ZDHHC9 expression may shape the BC immune microenvironment, alter tumor phenotypes, and impact antitumor immunity.

### 3.9 Prediction of ZDHHC9 on immunotherapeutic responses

**Figure 10:**
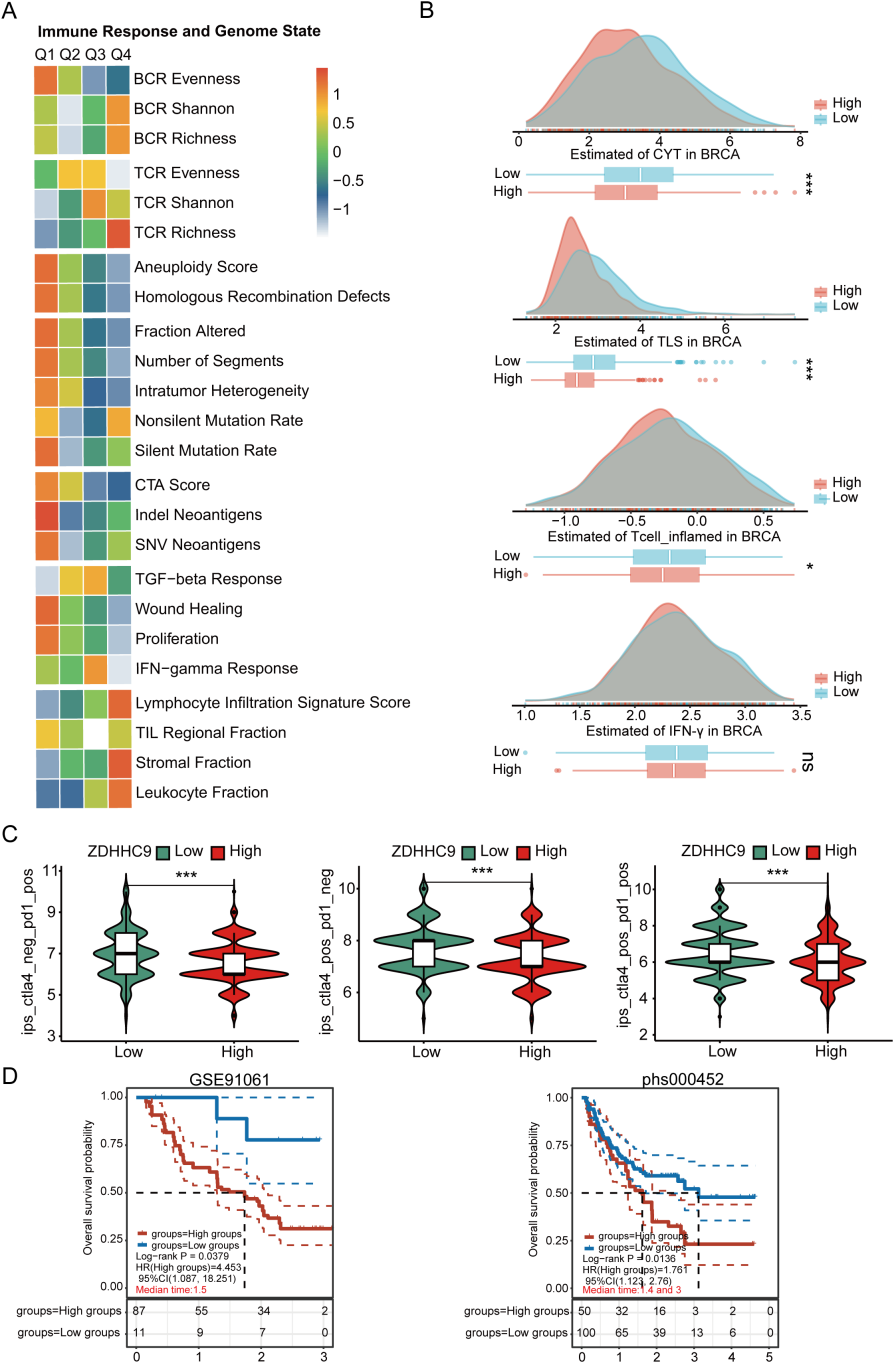
The relationship between ZDHHC9 expression and immunotherapy efficiency. **(A)** Heatmap showing the connections between ZDHHC9 expression and various immune indexes and genomic states. The heatmap represents the intra-group mean for each immune response score and genome status across Q1 to Q4 subtypes. **(B)** Differences in CYT, TLS, IFN-γ, and T cell_inflamed feature scores between high and low ZDHHC9 expression cohorts. **(C)** Analysis of ZDHHC9 expression with the efficacy of CTLA4 and PD-1 blocking therapies. **(D)** Survival analysis of the high and low ZDHHC9 patient groups in the two immunotherapy cohorts. (ns p≥0.05, *p < 0.05, ***p < 0.001).

To clearly understand the potential predictive value of ZDHHC9 in immunotherapy, various evaluation methods were employed. First, the correlation between ZDHHC9 expression and genomic features, along with immune-related indicators—including mutations, neoantigens, CTA (cancer testis antigens), and TCR/BCR diversity—was assessed. As depicted in **Figure 10A**, the Q1 cohort (characterized by high ZDHHC9 expression) demonstrated prominent mutation-associated attributes, such as Aneuploidy Score, Homologous Recombination Defects, Fraction Altered, Intratumor Heterogeneity, and so on, whereas the Q4 cohort (exhibiting low ZDHHC9 expression) exhibited enhanced TCR/BCR diversity, along with elevated Lymphocyte Infiltration Signature Scores, among other features. It has been reported that elevated levels of TILs correlate with favorable prognoses in BC (27). Subsequently, the EaSIeR model—a cancer-specific immune response model that functions as a biomarker-based tool to predict immunotherapy responses—was utilized. The CYT feature exhibits a positive connection with CD8^+^ T cells and cytokines, while the TLS feature shows a strong association with B cells. IFN-γ feature can be characterized by activated CD8^+^ T cells, and the T cell_inflamed feature integrates IFN-γ and inflammation-related T cell genes to predict responses to PD-1 blockade. The analysis suggested that subjects within the low ZDHHC9 expression cohort achieved higher scores for CYT, TLS, IFN-γ, and T cell-inflamed indicators, suggesting that these individuals might derive more benefit from immunotherapy (**Figure 10B**). Furthermore, IPS (Immunophenoscore), a recently identified predictive factor, was employed to evaluate patients’ potential responses to immunotherapy (28). The results revealed that the IPS scores were substantially higher in the low ZDHHC9 expression cohort compared to the high expression cohort (P < 0.001), reinforcing the notion that individuals with reduced ZDHHC9 expression might respond more favorably to immunotherapy (**Figure 10C**). Next, we conducted verification through two sets of immunotherapy data (GSE91061 and phs000452). The results revealed that patients in the low ZDHHC9 expression cohort showed better survival (OS) (**Figure 10D**). These findings underscore the utility of ZDHHC9 as a predictive indicator for ascertaining individuals with BC likely to benefit from immunotherapy.

### 3.10 ZDHHC9 expression predicts drug sensitivity

**Figure 11:**
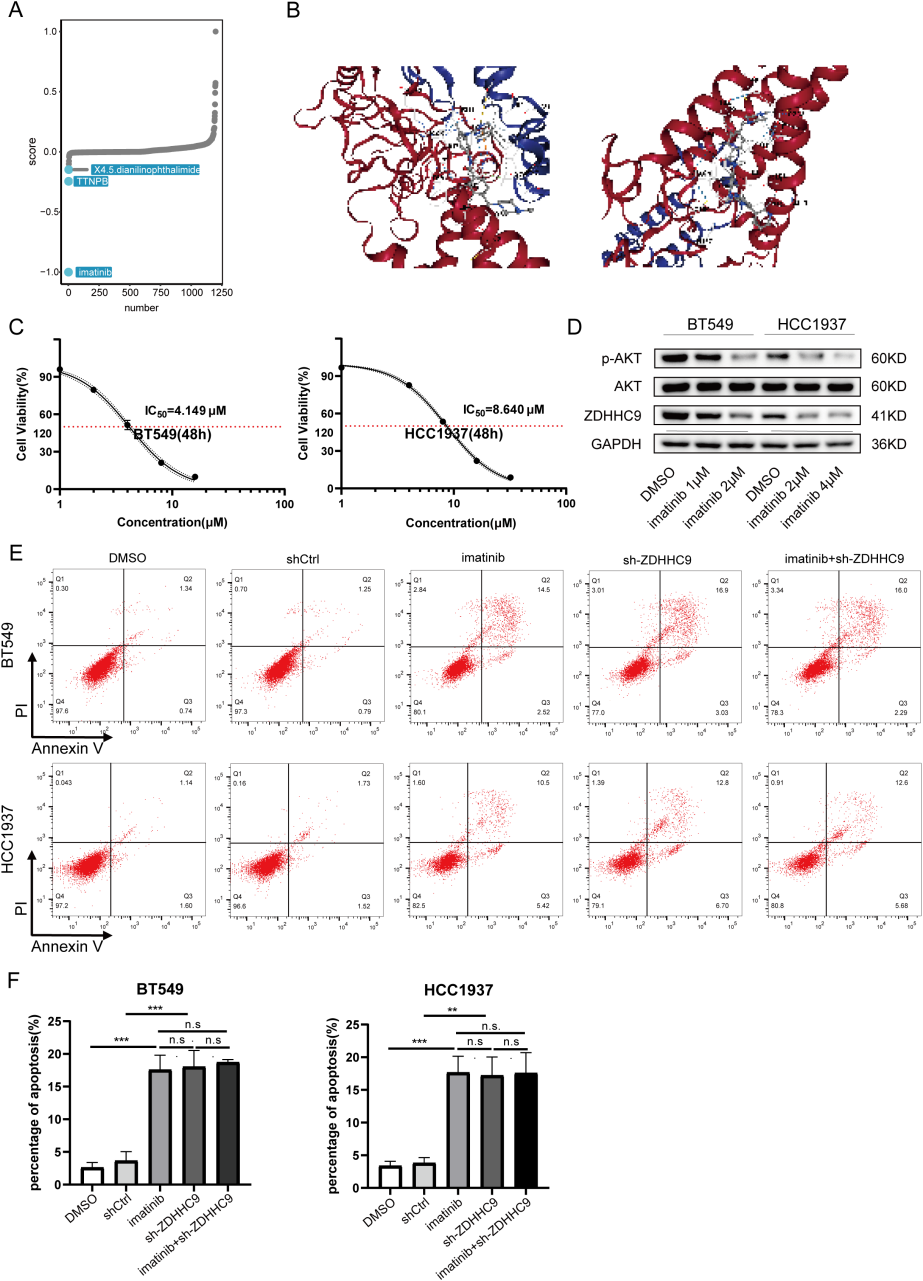
Drug sensitivity analysis of ZDHHC9. **(A)** Small-molecule drugs targeting ZDHHC9 identified from the CMap. **(B)** Molecular docking. Molecular docking between Imatinib and ZDHHC9 protein. **(C)** The IC50 of imatinib in BT549 and HCC1937 cells was determined by the CCK-8 assay. **(D)** After treating the BC cells with DMSO or imatinib for 48 hours, the expression levels of GAPDH, ZDHHC9, AKT and p-AKT in the cells were analyzed by western blotting. **(E, F)** Apoptotic rates were analyzed by flow cytometry in the following groups of BC cells (1): wild-type cells treated with DMSO or imatinib (2); cells transfected with empty vector or ZDHHC9-knockdown plasmids (3); ZDHHC9-knockdown cells treated with imatinib. Cells were exposed to imatinib (2 μM for BT549 and 4 μM for HCC1937) for 48 hours.” (**p < 0.01, ***p < 0.001).

Drug therapy remains an essential approach for treating BC. DEGs between cohorts with high and low ZDHHC9 expression were explored utilizing the CMap database, identifying three small-molecule drugs closely associated with ZDHHC9: X4.5.dianilinophthalimide, TTNPB, and Imatinib. Of these, Imatinib presented the lowest score, highlighting its potential to counteract the molecular effects induced by ZDHHC9 dysregulation and mitigate its oncogenic impact (**Figure 11A**). Subsequently, the three-dimensional structure of Imatinib was obtained from PubChem database, and the protein structure of ZDHHC9 (PDB ID: 8HF3) was obtained from RCSB PDB database, and molecular docking was performed through CB-DOCK2 database. **Figure 11B** showed the structure of protein-drug complex. Based on this, in order to explore the regulatory relationship between imatinib and ZDHHC9 in BC cells, we explored the IC_50_ values of imatinib in BT549 and HCC1937 cells through the CCK-8 assay (**Figure 11C**). Subsequently, the WB analysis revealed that imatinib downregulated the expression of ZDHHC9 in BC cells in a concentration-dependent manner, thereby inhibiting the phosphorylation of AKT (**Figure 11D**). In addition, flow cytometry experiments also found that compared with the DMSO group and the shCtrl group, both the ZDHHC9 knockdown group and the imatinib treatment group could induce apoptosis of BC cells, and the apoptotic ratios were comparable. However, in the ZDHHC9 knockdown +Imatinib treatment group, there was no significant difference in the proportion of induced apoptosis compared with the ZDHHC9 knockdown group or the Imatinib treatment group. This indicates that imatinib induces apoptosis and may exert its effect by down-regulating ZDHHC9 (**Figures 11E, F**).

### 3.11 ZDHHC9 catalyzes the palmitoylation of Akt in BC

**Figure 12:**
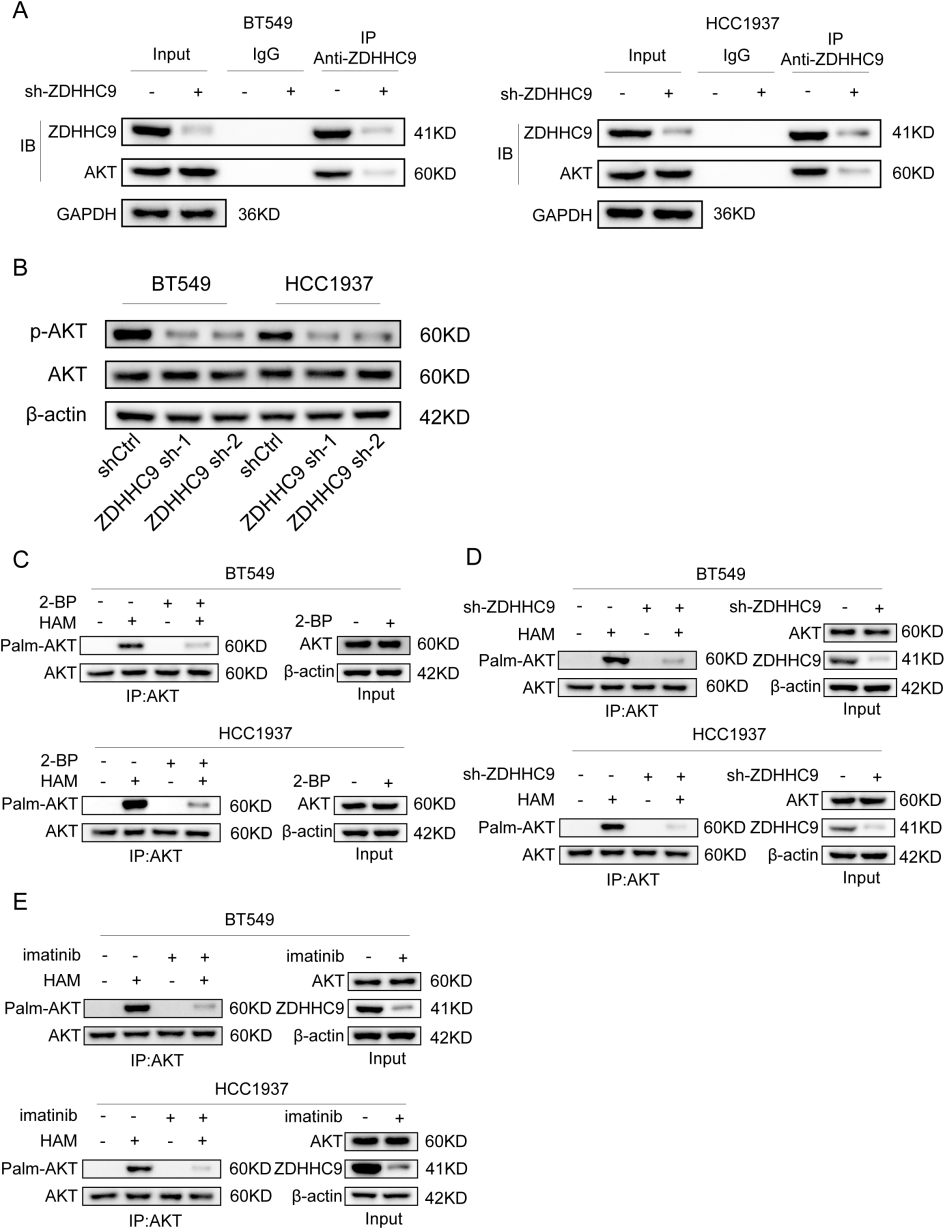
ZDHHC9 catalyzes the palmitoylation of Akt in BC. **(A)** The interaction between ZDHHC9 and AKT was demonstrated through the Co-IP assay. **(B)** Western blotting analysis of β-actin, AKT and p-AKT expression in control and sh-ZDHHC9 in BT549 and HCC1937 cells. **(C)** BT549 and HCC1937 cells were treated with 2-bromopalmitic acid (2-BP) at 50 µM for 12 hours. ABE determination was performed on the cells. **(D)** The palmitylation of AKT in BT549 and HCC1937 cells transfected with shCtrl or sh-ZDHHC9 plasmids was analyzed by ABE assay. **(E)** The palmitylation of AKT in BT549 and HCC1937 cells treated with DMSO or imatinib for 48 hours was analyzed by ABE assay. The concentrations of imatinib in treating BT549 and HCC1937 cells were 4µM and 8µM, respectively.

Based on the above results, the expression of ZDHHC9 is related to the tumor carcinogenic pathway. Moreover, a recent study reported that ZDHHC9-APT1-mediated Vangl2 stearoylation can regulate AKT signal transduction and thereby regulate the occurrence and development of tumors (29). For this purpose, we explored the relationship between the expression of ZDHHC9 and Akt. Firstly, the CO-IP experiment proved the interaction relationship between ZDHHC9 and Akt (**Figure 12A**). Subsequently, the effect of ZDHHC9 on Akt was investigated through Western Blotting experiments. The results showed that in BC cells, knockdown of ZDHHC9 could reduce the phosphorylation level of AKT. Inhibit AKT activation (**Figure 12B**). Subsequently, the acyl-biotin exchange method (ABE) was used to verify whether ZDHHC9 could catalyze the palmitoylation of Akt. The results showed that BT549 and HCC1937 cells treated with the broad-spectrum palmitoylation inhibitor 2-bromopalmitate (2-BP) could significantly reduce the palmitoylation of AKT. It indicates that AKT has the effect of S-palmitoylation through thioester bonds (**Figure 12C**). In addition, knockdown of ZDHHC9 also reduced the palmitoylation of AKT (**Figure 12D**). Furthermore, we also found that treatment with imatinib could also down-regulate the expression of ZDHHC9 and reduce the palmitoylation of AKT (**Figure 12E**).

## 4 Discussion

Our results indicate that ZDHHC9 is markedly overexpressed in BC tissues, and this overexpression is linked to more aggressive disease and worse patient outcomes. Consistently, recent studies in triple-negative BC (TNBC) have identified ZDHHC9 as a key factor associated with poor prognosis – TNBC patients with high ZDHHC9 levels exhibit shorter survival (30). High ZDHHC9 expression was also correlated with features of an immune-evasive tumor microenvironment (TME). Specifically, tumors with ZDHHC9 overexpression showed increased infiltration of immunosuppressive immune cells and upregulation of inhibitory checkpoints, alongside reduced expression of antigen-presenting machinery and pro-inflammatory chemokines (30). These findings suggest that ZDHHC9-rich tumors create an immunosuppressive TME or “cold” tumor phenotype. Indeed, analyses in other cancers reinforce this concept: in colorectal cancer, high ZDHHC9 was associated with worse survival and was shown to promote tumor growth by upregulating PD-L1 and blunting CD8^+^ T cell immunity (31). Likewise, in pancreatic cancer models, ZDHHC9 overexpression was linked to impaired anti-tumor immunity, whereas knocking down ZDHHC9 converted the TME from immunosuppressive to immune-active and improved treatment responses (32). Taken together, these data establish ZDHHC9 as a driver of an immune-tolerant milieu in multiple cancers, including BC, which likely contributes to its association with unfavorable clinical outcomes.

A novel insight from our single-cell RNA sequencing analysis is that ZDHHC9-overexpressing BC cells may shape the TME by engaging tumor-associated macrophages (TAMs) through the macrophage migration inhibitory factor (MIF) pathway. Ligand–receptor interaction mapping revealed that tumor-derived MIF and macrophage-expressed CD74 (with co-receptor CD44) form a signaling axis in ZDHHC9-high tumors. This paracrine MIF–CD74/CD44 interaction is a well-recognized mechanism of immunosuppression in the TME: MIF is often overexpressed in cancers and can drive macrophages toward an “M2” immunosuppressive, pro-tumoral phenotype (33). Notably, blocking the MIF–CD74 interaction has been shown to reprogram macrophages toward the pro-inflammatory “M1” state and enhance anti-tumor immune responses, for example by synergistically promoting microglia M1 polarization in lung cancer models (34). In line with these mechanisms, we observed that conditioned medium from ZDHHC9-overexpressing BC cells induced M2-like polarization of macrophages *in vitro*. These macrophages displayed high levels of prototypical M2 markers and immunosuppressive behavior. Strikingly, this effect was abrogated when ZDHHC9 was silenced or when tumor cells were treated with Imatinib, suggesting that ZDHHC9 activity in tumor cells is required for secreting factor(s) – likely including MIF – that skew macrophages to an alternative (M2) phenotype. By reversing this polarization, ZDHHC9 knockdown or pharmacologic inhibition can restore a more immunostimulatory TME. This aligns with the concept that targeting ZDHHC9 can relieve its immunoregulatory suppression and thereby potentially enhance T cell surveillance. Prior work demonstrated that palmitoylation of PD-L1 prevents its degradation, leading to increased PD-L1 on the tumor cell surface and augmented immune evasion (10, 35). Thus, by palmitoylating PD-L1 and fostering TAM-mediated immunosuppression via MIF, ZDHHC9 creates a multifaceted immune escape network within the TME.

Beyond its immunological effects, ZDHHC9 also exerts a direct pro-tumor effect by activating oncogenic signaling pathways. Our mechanistic data show that ZDHHC9 physically interacts with AKT and catalyzes the S-palmitoylation of AKT, which in turn promotes activation of the AKT pathway. Palmitoylation is the reversible attachment of a palmitate lipid to cysteine residues, often influencing protein localization and stability (36). We found that ZDHHC9-mediated palmitoylation of AKT enhances AKT membrane localization and boosts its phosphorylation, thereby upregulating downstream pro-survival signaling. It is noteworthy that AKT S-palmitoylation represents a novel layer of AKT regulation recently gaining recognition. For example, a 2024 study in hepatocellular carcinoma showed that the palmitoyltransferases ZDHHC17 and ZDHHC24 can palmitoylate AKT, anchoring it to the plasma membrane and significantly increasing AKT kinase activity (37). Disruption of AKT palmitoylation in that context, via either genetic means or pharmacological inhibitors of palmitoylation, led to reduced AKT signaling and tumor suppression. Given AKT’s central role in promoting cell proliferation, survival, and metabolism in BC, the ability of ZDHHC9 to activate AKT signaling provides a strong mechanistic basis for its oncogenic effects.

Immune checkpoint inhibitors (ICIs) have shown limited efficacy in BC overall – only a subset of patients, particularly those with “hot” tumors or triple-negative BC, experience durable benefits, while many do not respond (25). We found that patients with low ZDHHC9-expressing tumors had significantly higher immunophenoscore (IPS) and other immunogenic signature scores, indicating a more inflamed TME likely to respond to PD-1 or CTLA-4 blockade (**Figure 10C**). In line with this, analysis of two independent ICI-treated cohorts revealed that ZDHHC9-low patients exhibited better clinical outcomes under anti-PD-1/PD-L1 therapy (**Figure 10D**). These results are clinically meaningful – they suggest that ZDHHC9 might serve as a negative predictor of immunotherapy efficacy in BC, where low expression denotes a favorable immune contexture and higher chance of benefit from ICIs. Our conclusion is supported by parallel evidence in other cancers: In a recent pancreatic cancer study, ZDHHC9 silencing transformed the tumor milieu from “cold” to “hot” and dramatically sensitized tumors to anti-PD-L1 therapy *in vivo* (32). Likewise, inhibition of PD-L1 palmitoylation, which could be achieved by targeting ZDHHC9 or ZDHHC3, has been shown to boost T-cell killing of tumors and overcome immune resistance (35). It is noteworthy that palmitoylation inhibitors, such as 2-bromopalmitate or specific small molecules, are being explored for cancer therapy (38). Using the CMap databases, we found that high ZDHHC9 expression in BC was associated with increased sensitivity to certain chemotherapy agents and that the tyrosine kinase inhibitor imatinib scored highest as a potential repositioned drug to counteract ZDHHC9’s oncogenic effects (**Figure 11A**). Imatinib’s identification is intriguing – it could hint at crosstalk between ZDHHC9 and tyrosine kinase signaling or simply result from gene signature correlations.

Imatinib is a well-known tyrosine kinase inhibitor originally targeting BCR-ABL, c-KIT, and PDGFR which can effectively suppress ZDHHC9 and its downstream signaling. In our BC models, Imatinib treatment significantly downregulated ZDHHC9 expression and concomitantly reduced AKT phosphorylation. The inhibition of AKT activation by Imatinib is consistent with its known mode of action on upstream growth factor receptors; for instance, Imatinib has been shown to inhibit PDGFR-β in BC cells, leading to attenuation of PI3K/AKT signaling (39). We observed that Imatinib alone impeded BC cell growth, and notably, Imatinib may indirectly impair the palmitoylation of ZDHHC9’s substrates, such as AKT and PD-L1, thus phenocopying a direct ZDHHC9 inhibitor. Our findings align with a broader trend of targeting palmitoylation machinery as an anti-cancer strategy. In pancreatic cancer, for example, genetic silencing of ZDHHC9 was shown to sensitize tumors to anti-PD-L1 immunotherapy, effectively overcoming resistance to checkpoint blockade (32). While Imatinib is not a direct palmitoylation inhibitor, its capacity to modulate ZDHHC9 expression and AKT activity indicates a promising translational angle. Future development of specific ZDHHC9 inhibitors could provide even more potent means to shut down this pathway (40). Importantly, targeting ZDHHC9 is expected to have a dual impact: curbing tumor-intrinsic proliferation signals and mitigating tumor-induced immune evasion. This comprehensive understanding significantly strengthens the rationale for ZDHHC9 as a novel target for BC treatment and supports its prognostic value in identifying patients who might benefit from therapies counteracting tumor palmitoylation-dependent pathways (30).

## 5 Conclusion

In conclusion, elevated ZDHHC9 expression was not only associated with unfavorable outcomes in individuals with BC but also exhibited a negative correlation with several innate immune cells, suggesting that ZDHHC9 may suppress the antitumor immune response in BC. Thus, presents promise as both a survival indicator and a predictive indicator for immunotherapy in BC. However, several limitations must be acknowledged. Firstly, the differences in sequencing methods among different databases may introduce potential biases, thereby affecting the reliability. Secondly, our immunotherapy response analysis, though promising, was retrospective. The immunophenoscore and ICI cohort data we used serve as proxies for clinical response, but prospective clinical trials would be needed to verify that ZDHHC9-low tumors truly respond better to checkpoint inhibitors in practice. Additionally, patients in different ICI trials receive varied treatment regimens and combinations, these confounding factors could influence outcomes and were not fully accounted for. Thirdly, BC is a molecularly heterogeneous disease with intrinsic subtypes (Luminal A/B, HER2-enriched, basal-like, etc.) that we did not extensively stratify in our analysis. Our data suggested an overall trend of ZDHHC9 upregulation and poor outcome across BC, but subtype-specific differences remain to be explored. Finally, although the ESTIMATE and CIBERSORT algorithms were used to evaluate the microenvironment of ZDHHC9 in BC, its expression may still be disturbed by residual matrix RNA. In conclusion, despite these limitations, our study provides a compelling proof-of-concept that ZDHHC9 is integrally involved in BC tumorigenesis and immune regulation. By addressing the above gaps with further mechanistic experiments and subtype-specific investigations, future research can build on our work to fully realize ZDHHC9’s potential as a biomarker and therapeutic target in BC.

## Data Availability

The original contributions presented in the study are included in the article/**Supplementary Material**, further inquiries can be directed to the corresponding author/s.
