# Supplementary material for: Systematic analysis of ZDHHC9 as a potential prognostic and immunotherapy biomarker in breast cancer
Source: Front Immunol. 2025 Jul 16;16:1609621. doi: 10.3389/fimmu.2025.1609621 (PMC12307147; doi:10.3389/fimmu.2025.1609621)
Supplement: Supplementary file 1 [file DataSheet1.docx]

Supplementary Material

**Figure S1 Construction of Nomogram.**


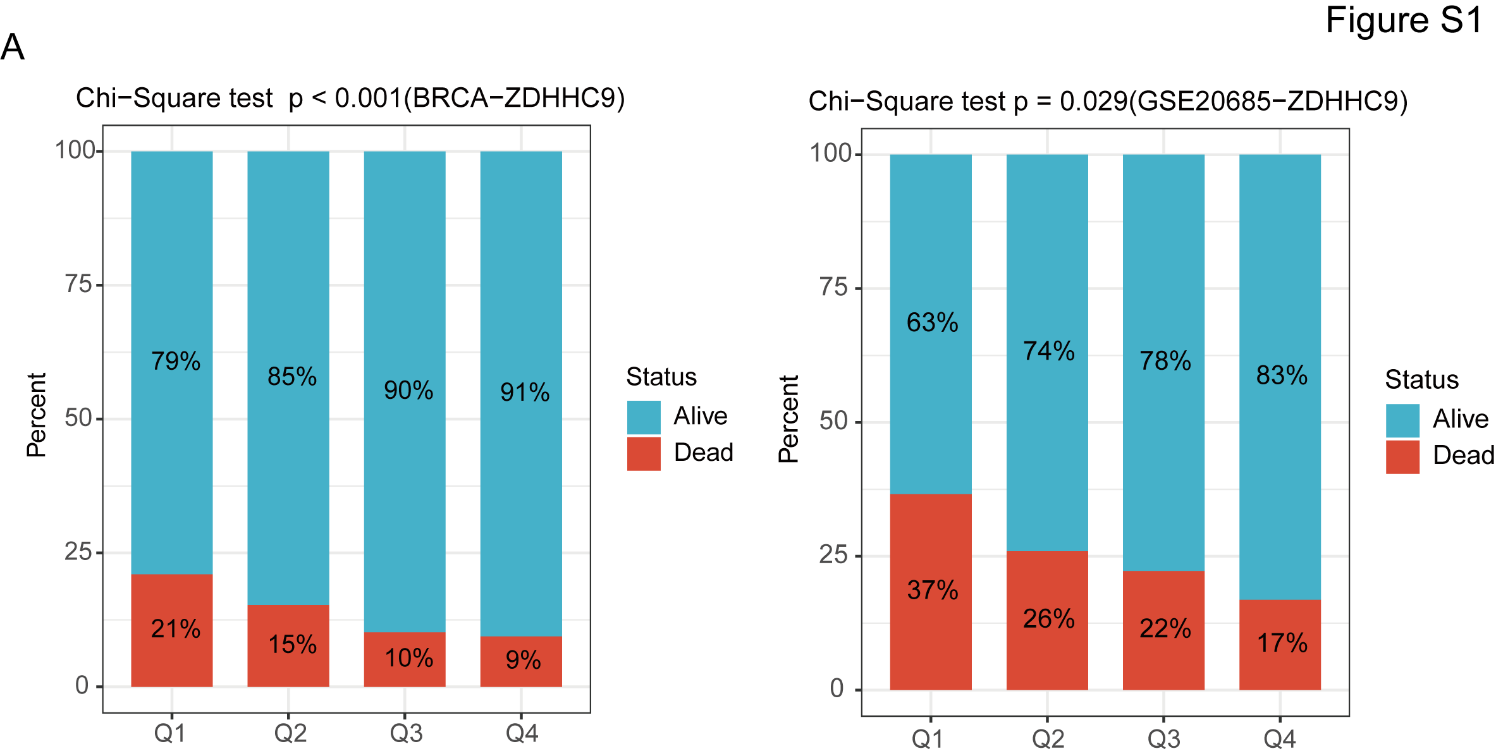


(A) The proportion of living and dead samples at different ZDHHC9 expression levels in the TCGA and GSE20685 datasets.

**Figure S2 Co-Expression Network of ZDHHC9.**

**
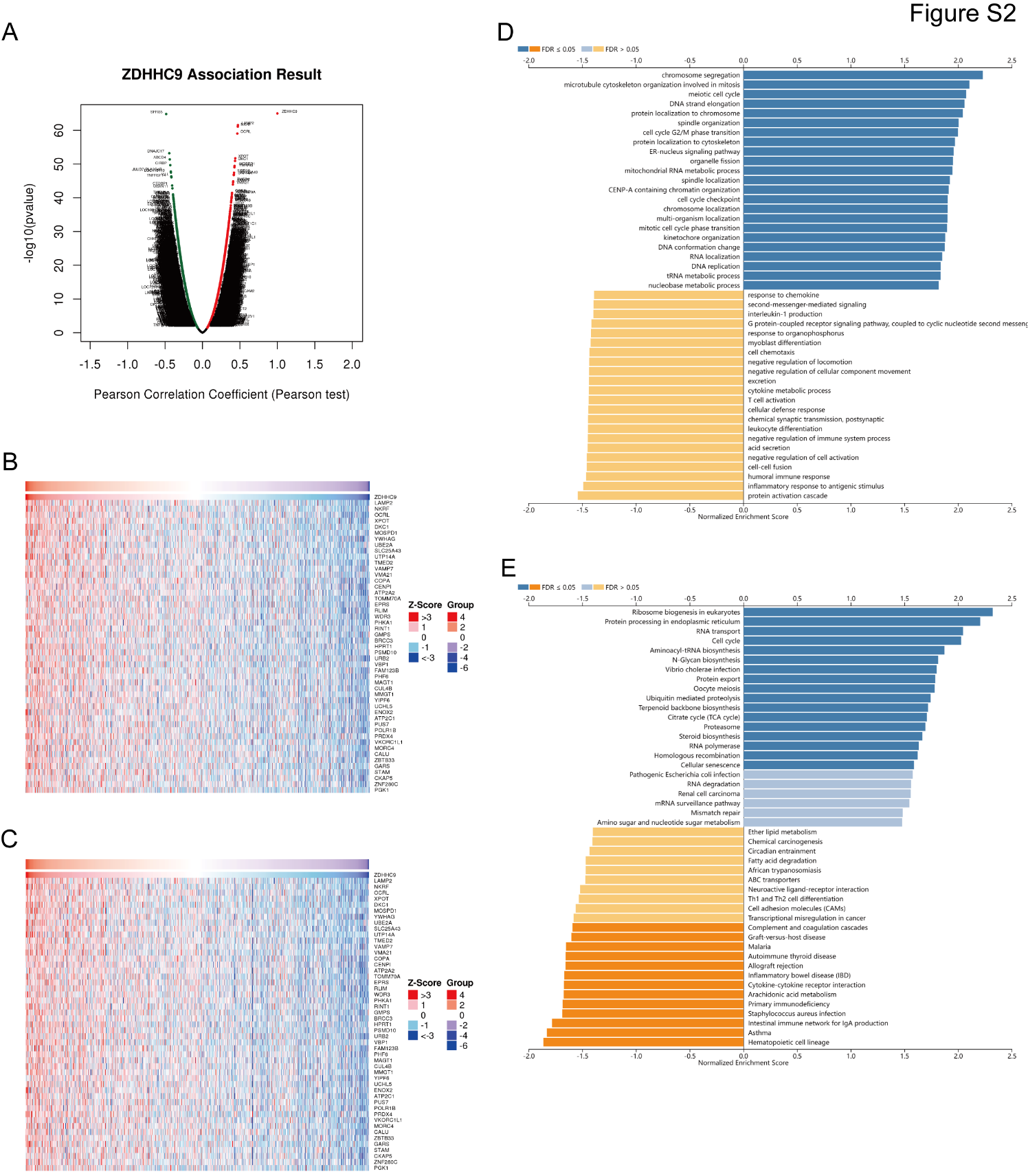
**

(A) Volcano plot indicating co-expressed genes correlated with ZDHHC9 expression in the TCGA-BRCA cohort. (B-C) Heatmaps depicting the top 50 co-expressed genes positively (B) and negatively (C) associated with ZDHHC9 expression in the TCGA-BRCA cohort. (D-E) GO_BP (D) and KEGG (E) analysis of co-expressed genes of ZDHHC9 in the BRCA cohort.

**Figure S3** **Functional Effects of sh-ZDHHC9 on BRCA Cells.**

**
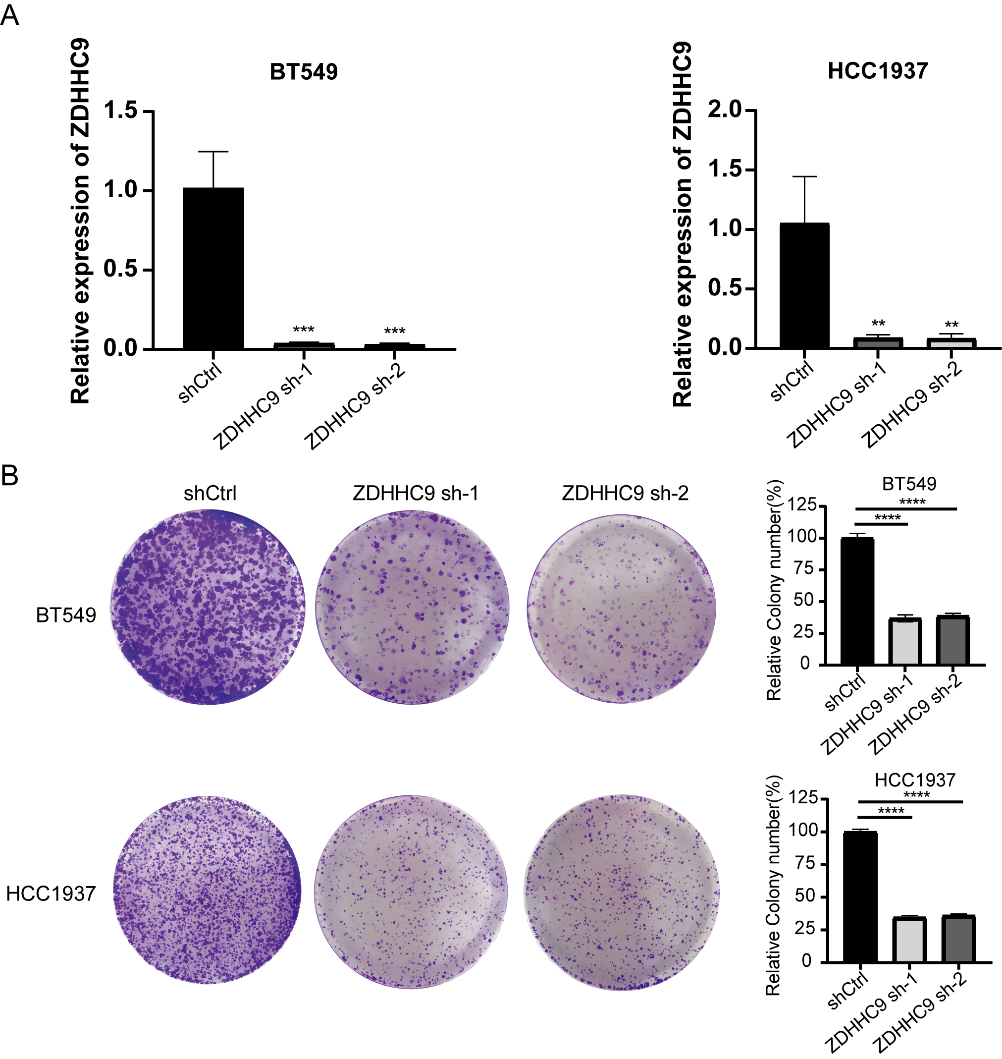
**

(A) RT- qPCR showed the efficacy of sh-ZDHHC9 in BT-549 and HCC1937 cells.

(B) Colony formation assay was used to assess the proliferation of BT-549 and HCC1937 cell lines.

# Figure S4 Immune landscape of ZDHHC9.


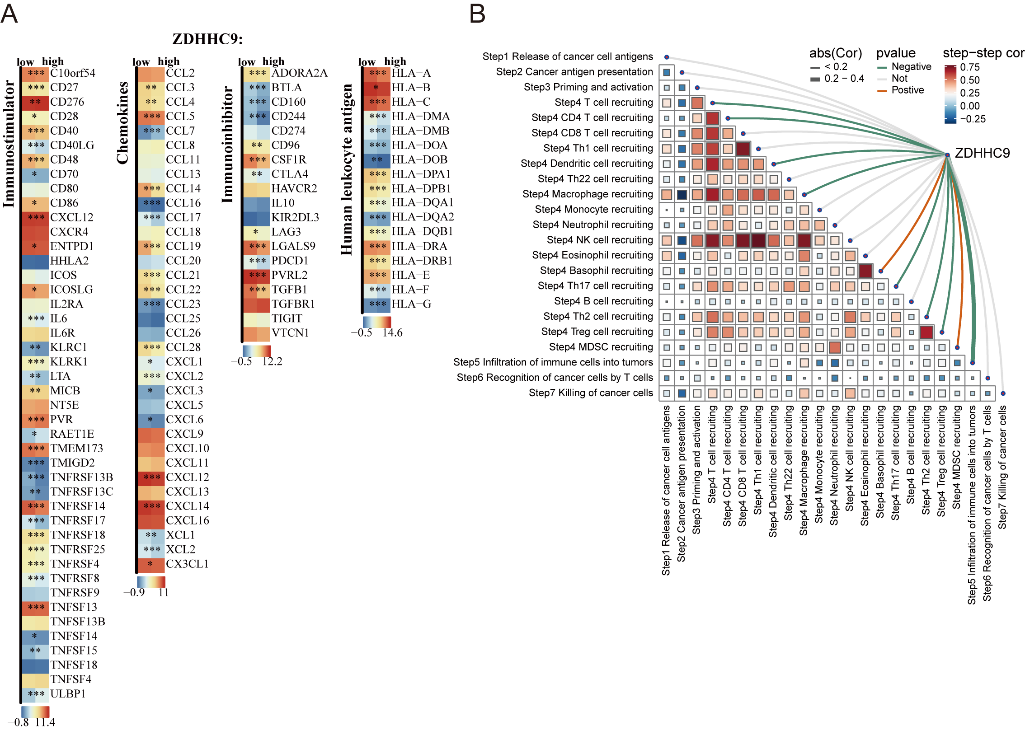


(A) Relationships between ZDHHC9 expression and immune modulators.

(B) Spearman correlation analysis between TIP score and ZDHHC9 expression. (ns p≥0.05, * p < 0.05, ** p < 0.01, *** p < 0.001).
